# Free-moving-state microscopic imaging of cerebral oxygenation and hemodynamics with a photoacoustic fiberscope

**DOI:** 10.1038/s41377-023-01348-3

**Published:** 2024-01-02

**Authors:** Xiaoxuan Zhong, Yizhi Liang, Xiaoyu Wang, Haoying Lan, Xue Bai, Long Jin, Bai-Ou Guan

**Affiliations:** Guangdong Provincial Key Laboratory of Optical Fiber Sensing and Communications, Institute of Photonics Technology, Jinan University, Guangzhou, 510632 China

**Keywords:** Imaging and sensing, Microscopy

## Abstract

We report the development of a head-mounted photoacoustic fiberscope for cerebral imaging in a freely behaving mouse. The 4.5-gram imaging probe has a 9-µm lateral resolution and 0.2-Hz frame rate over a 1.2-mm wide area. The probe can continuously monitor cerebral oxygenation and hemodynamic responses at single-vessel resolution, showing significantly different cerebrovascular responses to external stimuli under anesthesia and in the freely moving state. For example, when subjected to high-concentration CO_2_ respiration, enhanced oxygenation to compensate for hypercapnia can be visualized due to cerebral regulation in the freely moving state. Comparative studies exhibit significantly weakened compensation capabilities in obese rodents. This new imaging modality can be used for investigating both normal and pathological cerebrovascular functions and shows great promise for studying cerebral activity, disorders and their treatments.

## Introduction

The brain uses nearly a quarter of the body’s oxygen intake to support neural activities, and adequate oxygen delivery is essential for normal brain functions^1,2^. Oxygenation failure can induce cerebral hypoxia and may be fatal. Therefore, monitoring cerebral hemodynamics and oxygenation is critical for evaluating brain function, detecting brain injury^3^, and improving cerebral outcomes for intensive care medicine^4^. Rodents, e.g., mice, are the most commonly used animal models for neuroscience studies because of the functional similarities between rodent and human brains and their smaller size. However, benchtop microscopes can image brain activities only under anesthesia or with the head fixed. In contrast, head-mounted microscopes are desirable for free-moving-state cerebral imaging because brain activities can differ significantly in stationary and moving states^5^. In recent years, lightweight, head-mounted wide-field fluorescence microscopes^6^ and two-photon microscopes (TPMs) have been developed to image cerebral neural activity in freely behaving mice^7^. With an additional fluorescent agent, a TPM can be used to simultaneously image neural activity and the associated vasodilation to study neurovascular coupling^8^. However, these microscopes are not suitable for imaging the cerebral oxygenation process.

Photoacoustic microscopy, which is based on the detection of ultrasound waves induced by hemoglobin absorption with a focused laser, can be used to quantify blood oxygen saturation (sO_2_) and visualize the oxygenation and hypoxia in target tissue, i.e., a mouse brain, at single-vessel resolution^9–14^. The use of such microscopes for oxygenation imaging typically involves confocal alignment of lasers and ultrasound beams, rotational scanning with polygonal mirrors, and image stitching algorithms. These microscopes can be miniaturized and used on handheld probes; however, they are not suitable for wearable devices for small animals^15–19^. A possible strategy to achieve a head-mounted PAM is to use an unfocused transducer. For example, a head-attached PAM based on an unfocused piezoelectric sensor or a disposable polymer-based optical sensor, can be repeatedly used to view cerebrovascular structures^20,21^. Alternatively, single-pulse, wide-field cerebral imaging was achieved by using an ergodic relay cavity, in which the laser-induced ultrasound waves were reflected back and forth multiple times. The images were reconstructed based on the “fingerprint” waveform encoded in the temporal domain^22^. Nonetheless, these imaging modalities have yet to achieve the capability of imaging oxygenation and cerebrovascular responses in animals that are in a freely moving state.

Optical fiber technology offers versatile approaches for implementing miniaturized TPMs^23^, optical-coherence tomography (OCT) catheters^24^, and photoacoustic imaging devices^25^. Here, we report a photoacoustic fiberscope for high-spatiotemporal-resolution cerebral imaging in a freely behaving mouse. The imaging probe uses two optical fibers for photoacoustic excitation and detection, and the probe weight is only 4.5 grams. The scanner driving and sensor interrogation modules can freely rotate in compliance with the animal motion, allowing continuous cerebral imaging over the whole wakening process from anesthesia to the freely moving state. The fiberscope can be used to quantify the oxygen saturation (sO_2_), relative hemoglobin concentration (Hb), and vessel diameter associated with blood perfusion to assess cerebral oxygenation conditions. It has a 9-µm lateral resolution and 0.2-Hz frame rate, enabling the recording of cerebrovascular responses to external stimuli at single-vessel resolution. The photoacoustic fiberscope facilitates the monitoring of both normal and pathological cerebrovascular functions in freely moving animals, enabling advanced studies on diagnosis and treatment of cerebral disorders.

## Results

### The photoacoustic fiberscope

**Fig. 1:**
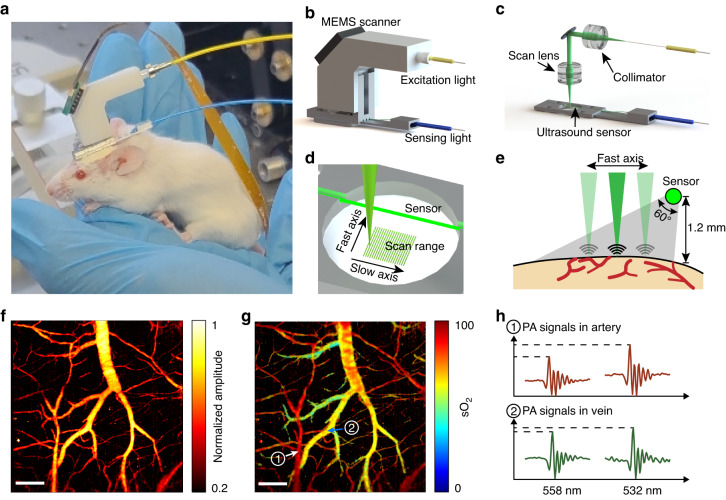
Photoacoustic fiberscope for cerebral imaging in the freely moving-state. **a** A freely moving mouse wearing the imaging probe. **b** External and (**c**) internal structure of the imaging probe. **d** Stereoscopic and (**e**) side views of the laser beam scanning and ultrasound detection schemes. **f** Photoacoustic images showing the relative hemoglobin concentration (Hb) and (**g**) oxygen saturation (sO_2_) in a selected region of the mouse cortex. **h** Photoacoustic signals after 558 and 532 nm excitation recorded in the artery and vein indicated in (**g**). The signals show different magnitude contrasts PA_532_/PA_558_ due to the different absorption spectra of oxy- and deoxygenated hemoglobin. Scale bar, 200 μm

Figure 1a shows a freely behaving mouse wearing an imaging probe, which was used to monitor cortical activity. The imaging probe uses two optical fibers for photoacoustic excitation and detection (Fig. 1b and c). The excitation laser beam is guided in the yellow-jacketed fiber (SMF-28e+, Corning), collimated with an achromatic lens (AC050-008-A, Thorlabs, N. A. = 0.33 in air), reflected by a 45-degree tilted mirror, and focused on the brain tissue by using an additional conjugated lens. These two identical lenses are used as a pair of relay lens with 1:1 amplification to project the laser light delivered in the optical fiber into the imaging plane. The hemoglobin in the cerebral vessels partially absorbs the laser pulses and generates ultrasound waves via the photoacoustic effect. A horizontally placed fiber optic sensor detects the laser-induced ultrasound waves. The blue-jacketed optical fiber guides the sensing light to the photodetector and signal demodulation module. Volumetric images are acquired via raster scanning of the laser beam using a microelectromechanical system (MEMS) scanner.

The imaging probe utilizes a fiber optic sensor to detect the photoacoustic signals. A small fiber laser with a lasing frequency of *ω*_0_ = ~2*π* × 195 THz is employed as the acoustically responsive element (see Methods). The laser cavity can be acoustically deformed, changing the lasing frequency by Δ*ω*_0_. The two orthogonally polarized laser modes have a 2-GHz frequency difference due to the intrinsic fiber birefringence. In the torsional-radial mode vibration state, the x- and y-polarized laser lights have identical frequency shifts but opposite signs, ±Δ*ω*_0_. We beat the two laser modes at radio frequency to collect the acoustically induced frequency change, 2Δ*ω*_0_. The radio-frequency beat signal responds to the ultrasound waves at *S* = 2.25 MHz kPa^-1^ over 30 MHz, which is mainly determined by the diameter, longitudinal and shear wave acoustic velocities and photo elastic coefficient of the optical fiber. The sensor can offer a noise-equivalent pressure (NEP) of 8 Pa.

Figure 1d, e show the laser-beam scanning and ultrasound detection schemes (see Fig. S1, Fig. S2 and Fig. S6 for details). The fiber optic sensor is immersed in an acoustic coupling medium. The sensor is placed 1.2 mm above the tissue surface, is parallel with the imaging plane, and detects ultrasound waves in a side-viewing manner. The sensor has a sensitive length of ~3 mm, which is defined by the two highly reflective Bragg reflectors. The ultrasound response has a cos(2*θ*) dependence, where *θ* denotes the incident angle of the ultrasound wave, offering a 60-degree view angle at half magnitude. By rotating the sensor, the overlap between the laser scanning and ultrasound detection areas can be maximized (see Fig. S6). The optical window for the excitation laser beam is sealed with a glass coverslip. The unfocused ultrasound detection reduces the optical alignment requirements, allowing an A-line scan rate of 100 kHz, a B-scan rate of 100 Hz, and a frame rate of 0.2 Hz over a 1.2 mm by 1.2 mm area. As a result, the imaging probe enables fast scanning, high optical resolution imaging of cerebral hemodynamics.

The headpiece has an aluminum baseplate (weight: 0.1 gram), which is permanently fixed on the mouse head over the cranial window. The location of the cranial window is shown in Fig. S1. It is noteworthy that the applied stimuli in the context, for example, CO_2_ or N_2_ respiration, elicits a systematic change in cerebral hemodynamics and oxygenation, and our study did not intentionally focus on specific brain regions. The baseplate contains four permanent magnets in its corners, which are used to mount the imaging probe (see Fig. S1). The imaging probe was assembled by incorporating the optical fibers, lenses, and MEMS scanner before attaching the probe to the baseplate (see Fig. S2). This design is convenient for mounting the probe and guarantees imaging stability in freely behaving mice. Furthermore, this detachable design allows repeated imaging of the region of interest (ROI) based on the experimental requirements.

The experimental mouse wearing the imaging probe was placed in a cylindrical chamber that allowed free behavior (see Fig. S3). We mounted a camera on the top of the chamber to track and record the motion of the mouse. The chamber has a gas inlet and an outlet at the bottom for hypercapnia and hypoxia experiments. The imaging probe is connected to the console through two optical fibers and an electrical wire. To prevent wire/fiber entanglements, the scanner driving, and sensor interrogation unit was miniaturized, with an overall volume of 11×12×1.7 cm^3^, which includes the 980-nm semiconductor laser pump source, erbium-doped fiber amplifier, photodetector, fiber polarizer, and optical isolator. A coaxial optical/electrical slip ring was used to transmit the excitation laser beam, supply power, and acquire electrical signals. As a result, this unit can rotate freely and is compliant with the motion of the experimental animal, allowing continuous cerebral imaging. The freely moving mouse can bend and vibrate the optical fibers in the experiment. However, the two orthogonally polarized laser beams of the sensing laser are highly correlated, and the heterodyne detection scheme utilizes common-noise cancelation to stabilize the sensor output during cerebral imaging. The lengths of the optical fibers were optimized to adapt to the size of the chamber.

For oxygenation imaging, we built a customized dual-colored laser source (see Methods and Fig. S4). The 558-nm component is produced by the second-order Stokes wave generated by the stimulated Raman scattering in the highly nonlinear optical fiber. This component was combined with a 532-nm laser beam with the same repetition rate and an optimal pulse interval of 2.75 µs before being input into the imaging probe. A field-programmable gate array card (PXI-7852R, National Instruments) was used to synchronize the laser pulses, the MEMS scanner, and the data acquisition module. We use a LabVIEW program to acquire and process data. Figure 1f, g demonstrate photoacoustic images showing cerebral hemoglobin and sO_2_ in the same region. A depth calibration was performed to compensate for the varying time-of-flight of photoacoustic signals over the imaged area before reaching the ultrasound sensor (see Fig. S6). Small veins, arteries, and capillaries can be visualized and distinguished in the images. Oxy- and deoxygenated hemoglobin have different absorption coefficients at these two wavelengths. Identical laser energies induce photoacoustic signals with different strengths PA_532_ and PA_558_ (Fig. 1h). As a result, oxygen saturation (sO_2_), namely, the molecular ratio of oxygenated hemoglobin to total hemoglobin, can be quantitatively measured and imaged based on Eq. (1) (see Methods). The lateral resolution is measured as 9.6 µm (see Fig. S5a), corresponding to a 0.03 numerical aperture of the imaging system, which is determined by mode field diameter the optical fiber. The axial resolution is estimated as 165 µm (see Fig. S5b), limited by the mismatch in acoustic impedance between the fiber glass and water.

### Hypercapnia experiment under anesthesia

**Fig. 2:**
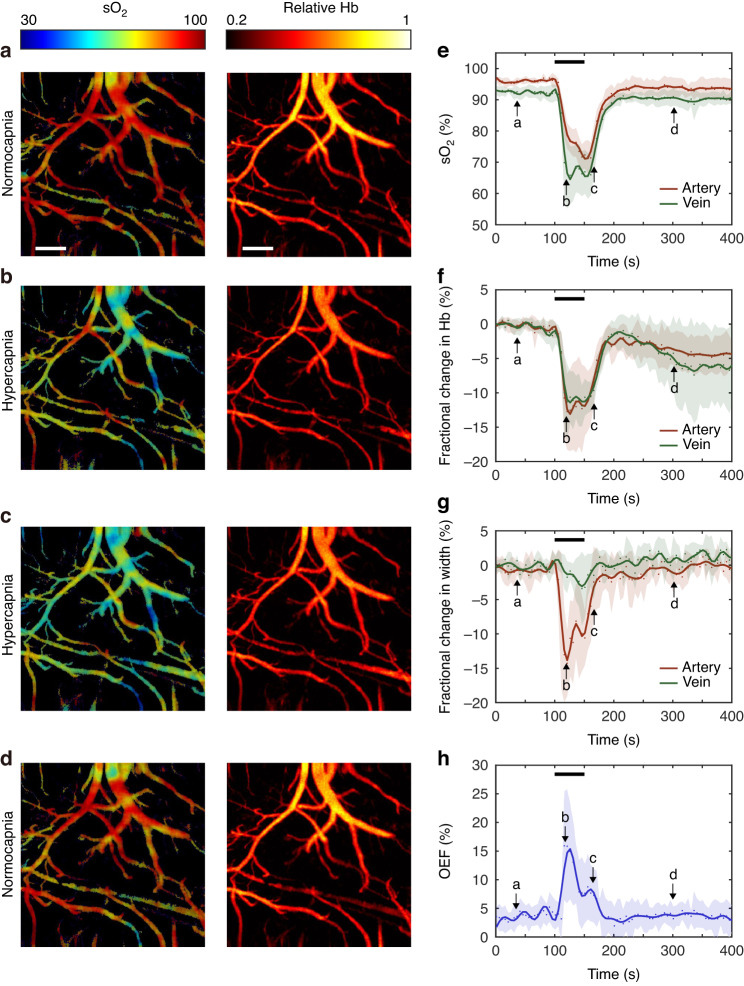
High-concentration CO_2_ respiration induced cerebrovascular responses under anesthesia. **a**–**d** Hb and sO_2_ photoacoustic images in a normocapnia-hypercapnia cycle. Recorded variations in sO_2_ (**e**) Hb (**f**) vessel diameter (**g**) and oxygen extration fraction (OEF) (**h**). *n* = 4. The data in (**e**–**h**) are shown as the mean ± s.e.m. The arrows in (**e**–**h**) show the transients corresponding to (**a**–**d**). The black lines indicate the period of the hypercapnia experiment. Scale bar, 200 μm

We first investigated acute hypercapnia in the experimental animal under anesthesia and performed cerebral imaging with the photoacoustic fiberscope. We performed baseline imaging under normocapnia conditions (Fig. 2a). Then, we changed the respiratory gas from normal air (with 1.5% isoflurane anesthesia) to a 50%:50% air/CO_2_ mixture to induce hypercapnia. A high CO_2_ concentration was used to generate a quick cerebrovascular response (Supplementary Video 1). After imaging, we repeated the hypercapnia experiment and performed a cardiac blood analysis 50 s after the animal was presented with the air/CO_2_ mixture. The measured CO_2_ partial pressure of 100 ± 12 mmHg (normal condition: 40 mmHg) and oxygen partial pressure of 34 ± 8 mmHg (normal state: 90 mmHg) verified the occurrence of acute hypercapnia. As depicted in Fig. 2b, c, hypercapnia induced a significant reduction in both the overall oxygen saturation (sO_2_) and the relative hemoglobin concentration (Hb). After the hypercapnia experiment, the sO_2_ and Hb levels recovered to normal levels (Fig. 2d).

We performed normocapnia–hypercapnia imaging with four mice and extracted the changes in the sO_2_ and Hb levels and the diameters of the veins and arteries (Fig. 2e–g, see Fig. S7 for extraction of sO_2_/Hb/vessel diameter variations from the imaging result). Figure 2e shows that the averaged venous sO_2_ level decreased from 92% to 64%, and the arterial sO_2_ level decreased from 95% to 75% in the first 10 s before decreasing to a minimal value of 70% (see Fig. S9 for the statistical results). Figure 2f exhibits that the Hb level decreased in both the arteries and veins by ~10%. Figure 2g shows the vasoconstriction in an artery (by 15%) and a vein (2.5%). sO_2_ and Hb recovery took 50 s. These results are consistent with previous measurements of high-concentration CO_2_ respiration in animals^26^. Moreover, the oxygen extraction fraction (OEF), which is defined as the ratio of oxygen consumption to delivery in a tissue or organ, is a useful parameter for quantifying abnormal cerebrovascular oxygenation. Figure 2h shows the OEF variation in the hypercapnia experiment, which was calculated based on the sO_2_ measurement results (see Methods). The OEF increased from 5% to 15% in the hypercapnia experiment due to insufficient oxygen delivery.

We then imaged the cerebrovascular responses to lower CO_2_ concentrations, by replacing normal respiration air with a 90%:10% air/CO_2_ mixture, and this experiment was repeated with five mice and lasted for 300 s (see Fig. S10). In contrast to the response to 50% CO_2_ respiration, 10% CO_2_ respiration resulted in ~25% vasodilation and an Hb increase of ~14% in the arteries. Additionally, it induced an increase in venous sO_2_ from 79% to 89%. These results were consistent with previous literature^27–29^ and confirmed using a benchtop stereomicroscope (refer to Fig. S8 and Fig. S10). It is noteworthy that cerebral vessels exhibit vasodilation and vasoconstriction in response to 10% and 50% CO_2_ respiration experiments, respectively. The contrasting responses to 10% and 50% CO_2_ respiration are not yet fully understood. However, it is plausible that high concentrations of CO_2_ may induce significant alterations in animal circulation, such as blood pressure, while lower CO_2_ concentrations may predominantly affect microcirculation.

We also conducted a N_2_ hypoxia experiment (see Fig. S11 and Supplementary Video 2) on anesthetized mice for comparison. Here, we reduced the oxygen fraction from ~21% of the respiratory air to 10% by mixing equal volumes of air and nitrogen. This experiment lasted for 100 s and was repeated with five mice. Results showed that hypoxia induced a decrease in sO_2_ from 90% to 70% in the veins and 95% to 80% in the arteries, as well as a decrease in Hb of ~7% (refer to Fig. S12 for statistical results). Moreover, during the hypoxia experiment, we observed a maximum of 3% vasodilation in the vein and a change in OEF in the cerebral vessels from 4% to 8%. These hemodynamic responses are consistent with previous medical studies and imaging results obtained using benchtop photoacoustic microscopy^10^.

### Cerebral imaging in the wakening process

**Fig. 3:**
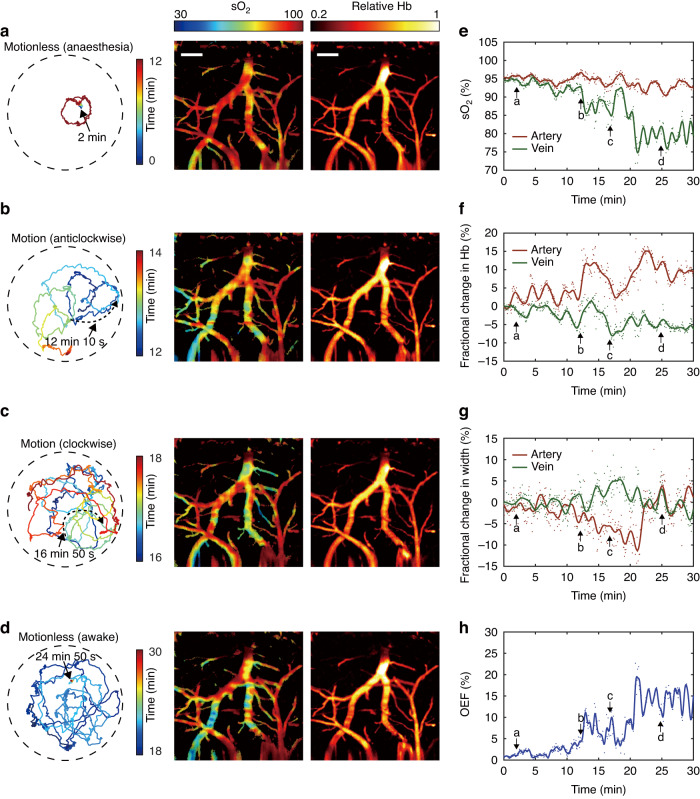
Cerebral imaging in the awakening process over 30 min **a**–**d** Hb and sO_2_ photoacoustic images of the cortex during four selected periods. The motion trajectories over each period are also shown. The pseudo color scale of each trace line represents the time. **e** sO_2_, (**f**) Hb, (**g**) vessel diameter, and (**h**) OEF changes in the arteries and veins. Scale bar: 200 μm. Dots in (**e**–**h**): raw data; solid curves in (**e**–**h**): lowpass filtered result

Next, we placed an isoflurane-anesthetized, 8-week-old male BALB/c mouse wearing an imaging probe on its head in the chamber and continuously imaged its cerebral activity. Supplementary Video 3 shows the recorded mouse behavior during the period from anesthesia to freely moving and the real-time sO_2_ and Hb photoacoustic images over 30 min. The video shows that the fiberscope can stably image cerebral hemodynamics in a freely behaving state, even when the mouse accidentally hits the chamber wall. Figure 3 demonstrates the motion trajectory of the mouse during wakening (Fig. 3a), anticlockwise movement (Fig. 3b), clockwise movement (Fig. 3c), and a short rest period in the chamber (Fig. 3d, see Methods for details about the motion trajectory tracking). The four photoacoustic images presented in Fig. 3a–d show that the sO_2_ levels in the veins decreased when the mouse woke and moved freely. In the first 20 min, venous sO_2_ decreased (Fig. 3e), arterial Hb increased (Fig. 3f) and vasoconstriction (Fig. 3g), and OEF increased (Fig. 3h) due to the increased oxygen metabolism in the wakening and freely moving states and the reduced isoflurane anesthesia. After 20 min, cerebral oxygenation recovered to a normal state. The motion-induced bending and vibration of the fiber may cause fluctuations in the excitation pulse energy at the cortex. This issue can be addressed by using a large-core single-mode photonic crystal fiber for slip-ring fabrication to improve stability. Despite the motion noise, the fiberscope can still capture cerebral hemodynamic changes.

### Hypercapnia experiment in freely behaving mice

**Fig. 4:**
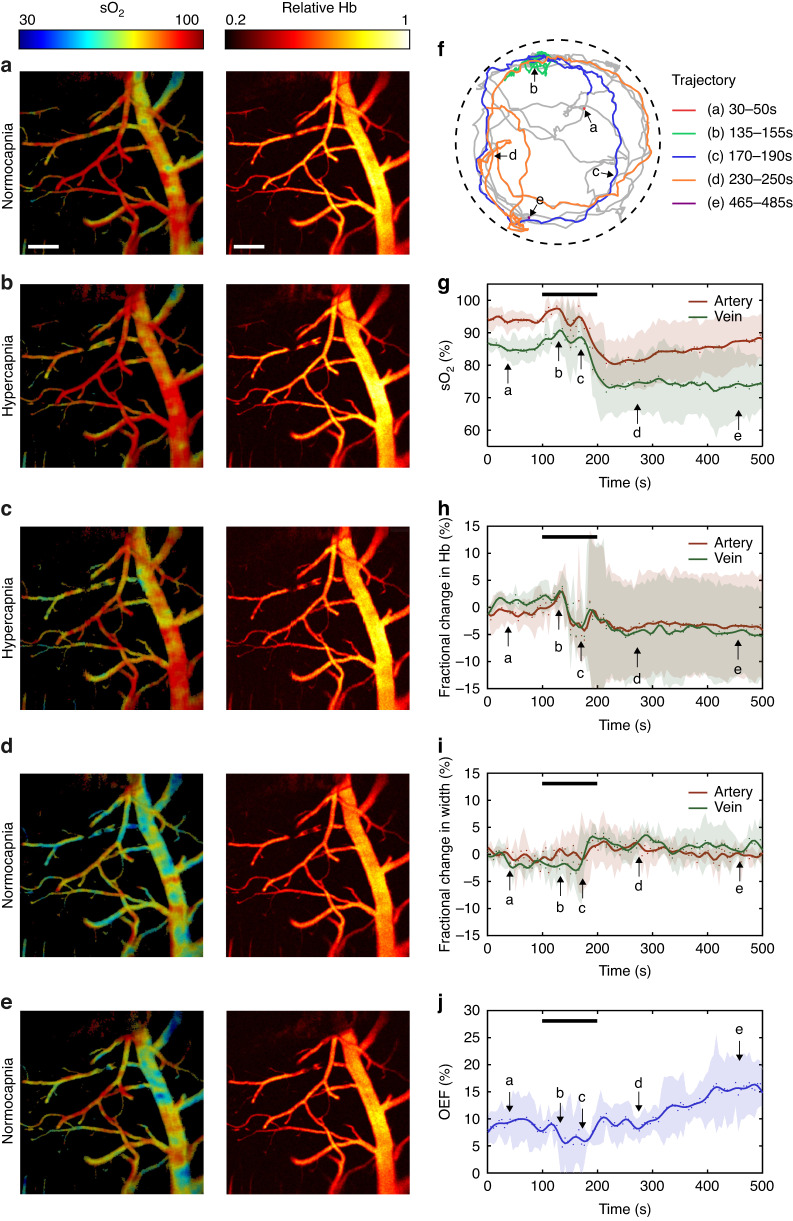
Hypercapnia experiment in freely behaving mice. **a**–**e** Hb and sO_2_ photoacoustic images in freely moving mice in a hypercapnia-normocapnia cycle. **f** Motion trajectories recorded during the experiment. **g** sO_2_, (**h**) Hb, (**i**) vessel diameter in the arteries and veins, and (**j**) Oxygen extraction fraction (OEF) change in the region of interest. The black lines in (**g**–**j**) represent the period of hypercapnia. The arrows represent the time transients corresponding to (**a**–**e**). *n* = 4. The data in (**g**–**j**) are shown as the mean ± s.e.m. Scale bar, 200 μm

Next, we imaged hypercapnia-induced cerebral responses in a freely moving mouse (Supplementary Video 4). The experiment was performed over 100 s. Figure 4 shows the sO_2_ and Hb photoacoustic images captured during the baseline (Fig. 4a), hypercapnia (Fig. 4b, c), and normocapnia states (Fig. 4d, e). The imaging results show that the overall sO_2_ level increased slightly when CO_2_ was injected into the chamber and subsequently decreased after the experiment. The mouse could move freely in the chamber during the hypercapnia experiment, as shown by the recorded motion trajectory in Fig. 4f. We repeated the imaging experiment with four healthy mice to record the temporal variations in the individual hemodynamic parameters. Figure 4g–j demonstrate that the sO_2_ level increased (by 5% in the artery and 4% in the vein, Fig. 4g), Hb levels slightly increased (Fig. 4h), vasodilation decreased (by 5% in the vein and 2% in the artery, Fig. 4i), and the OEF decreased by 4% (Fig. 4j). The results suggest that the cerebral vessels tend to deliver more oxygen to compensate for hypercapnia. This oxygenation enhancement induced by CO_2_ respiration was not observed in the anesthetized mice. As shown in the motion trajectory in Fig. 4f, this compensation process is accompanied by fast mouse locomotion (frames in c and d). As the hypercapnia continued, the arterial and veinous sO_2_ levels decreased by 15% and 14% (beginning at ~150 s), and the total Hb level decreased by ~5%. The OEF gradually increased over the next 300 s, corresponding to insufficient blood oxygen delivery, which is consistent with previous observations in human experiments conducted under hypoxic conditions^30,31^. To assess the impact of the weight of the head-mounted fiberscope on mouse behavior, we recorded the motion trajectories of three adult male mice (weights: 28–30 g) with and without wearing the headpiece and wire connections. The mice were allowed to move freely in a 40-cm-wide region for 30 min. The measured results indicate that there were minimal changes in mouse moving distance and speed (refer to Fig. S13).

**Fig. 5:**
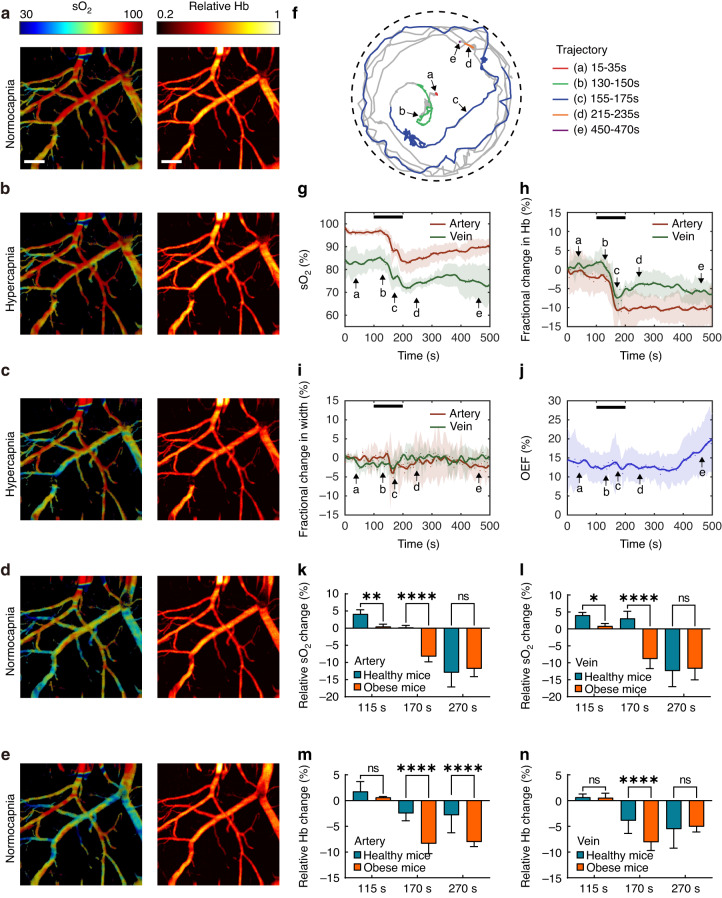
Hypercapnia experiment in freely behaving obese mice. **a**–**e** Hb and sO_2_ photoacoustic images in freely moving mice in a hypercapnia-normocapnia cycle. **f** Motion trajectories recorded during the experiment. **g** sO_2_, (**h**) Hb, (**i**) vessel diameter in the arteries and veins, and (**j**) Oxygen extraction fraction (OEF) change in the region of interest. Comparison of changes in (**k**) sO_2_ in the artery, (**l**) sO_2_ in the vein, (**m**) Hb in the artery, and (**n**) Hb in the vein relative to the baseline during the experiment between healthy and obese mice. The black lines in (**g**–**j**) represent the period of hypercapnia. The arrows represent the time transients corresponding to (**a**–**e**). The data in (**g**–**j**) are shown as the mean ± s.e.m. Here, the *P*-values were determined by two-way ANOVA. *P < 0.05, **P < 0.01, ****P < 0.0001, ns not significant (healthy mice *n* = 4, obese mice *n* = 4). Scale bar, 200 μm

Hyperlipidemia is known as one of the major causes of vascular dysfunction^32,33^. It can slow blood flow, cause cerebral hypoperfusion, and induce chronic brain hypoxia^33–36^. Here, we also imaged the cerebrovascular responses in freely moving obese (hyperlipidemia) mice (Fig. 5 and Supplementary Video 5, see Methods for details). The photoacoustic images shown in Fig. 5a–e demonstrate that the overall sO_2_ levels tended to remain stable (Fig. 5b, g) and then decreased by ~10% during the experiment (Fig. 5c, g). In addition, the Hb levels decreased by ~10% in the arteries and ~5% in the veins (Fig. 5h). The vessel diameter increased by ~2% in the vein and decreased by ~2% in the artery (Fig. 5i). After the experiment, the sO_2_ levels in the cerebral veins remained low. The tracked motion trajectory of one of the experimental mice presented in Fig. 5f shows that the obese mice can freely move in the experiment. We selected three periods and compared the cerebral responses of the healthy and obese mice, including period #1 (115–135 s, at the beginning of the hypercapnia experiment), period #2 (170–190 s, at the end of the experiment), and period #3 (270–290 s, after the experiment). The statistical results shown in Fig. 5k–n were calculated based on the measured results presented in Figs. 4 and 5 over the three selected periods. The results suggest that oxygenation enhancement occurred in both the healthy and obese groups. However, the induced oxygenation was significantly stronger in the control group than in the obese group (Fig. 5k, l). The Hb levels in the veins and arteries decreased significantly 70–90 s after gas injection.

The different hypercapnia-induced responses in obese and healthy mice can be compared by analyzing the results presented in Figs. 4 and 5. For instance, the OEF decrease for the control group was hardly observable in the obese group. Moreover, the sO_2_ level decreased at 120 s (20 s after CO_2_ injection), which was earlier than in the control group. Additionally, the baseline OEF values in obese mice were higher than those in healthy mice. Based on our measurements, it appears to take ~30 min for mice to recover to their baseline state. However, it is important to acknowledge that different mice may have varying recovery durations, which can lead to a significantly expanding error bars in individual measurements. In this study, we mainly focused on fast hemodynamic responses and therefore did not record long-time traces.

To ensure consistency, we carefully controlled the experimental conditions for cerebral imaging of healthy and obese mice, including the imaged cortical area, gas concentration, and respiration duration. In addition, we recorded the motion trajectories of four healthy and four obese mice during the CO_2_ respiration experiment (lasting between 100 and 200 s, see Fig. S14). The results indicated that there was no significant difference in moving distance or speed between healthy and obese mice during the experiment. Regarding movement noise, we conducted experiments on multiple mice and found that the phenomenon of compensated oxygenation was consistent and reproducible, while the motion trajectory did not exhibit any temporal relevance.

## Discussion

In conclusion, we developed a photoacoustic fiberscope to visualize cerebral hemodynamics and oxygen metabolism in freely behaving rodents at single-vessel resolution. This imaging modality can be used to visualize the different cerebrovascular responses of anesthetized and freely behaving mice. In the freely behaving mice, we observed an immediate increase in oxygen supply after high-concentration CO_2_ respiration, which was not present in anesthetized mice. Furthermore, the results of comparative studies showed that the oxygen compensation capability is notably weaker in obese mice compared to that in control mice.

For comparison, we employed a benchtop stereomicroscope as a benchmark measurement in conjunction with our photoacoustic fiberscope to monitor cerebrovascular responses to high (50%) and low (10%) CO_2_ respiration concentrations (see Fig. S8 and Fig. S10). The observed hemoglobin changes and vasodilation/vasoconstriction phenomena were consistent across both methods. Nevertheless, the stereomicroscope demonstrated lower imaging contrast and lacked sO_2_ measurement functionality.

We observed an upregulation in the venous sO_2_ levels in the mouse brain under isoflurane by using the photoacoustic fiberscope. This was consistent with prior literature, indicating that venous sO_2_ levels can escalate to between 85% and 90% under isoflurane^37,38^, even at lower oxygen concentrations (our experiment used 20% compared to the 12% documented in the literature^37^). We found that the baseline venous sO_2_ levels in awake mice were significantly higher than those documented in previous literature, ranging from 70% to 85%^37,39,40^. This discrepancy is likely attributable to the absence of a 1-day or 5-day habituation process^37,40^. The shorter adaptation and recovery period we provided following skull removal may have induced stress, which could have increased blood oxygenation^38^.

As evidenced by the quantified results in Figs. 2, 4, and 5, our fiberscope can measure sO_2_/Hb changes with an error of <5% in the anesthetized state and 10% in the freely moving state for multiple mice monitoring. This precision enables us to visualize cerebrovascular responses induced by hypoxia or hypercapnia. Furthermore, strong stimuli can induce a sO_2_ change exceeding 10%, which is detectable using the fiberscope under neurovascular couplings. However, for detecting weaker stimuli or visualizing functional couplings across different brain functional areas in a resting state, further enhancements in measurement capability may be required.

The cerebrovascular responses of head-fixed mice have been studied by using multiple technological means. For example, the cerebral oxygen partial pressure (pO_2_) has been measured using Clark-type polarographic electrodes^41^. Additionally, spectroscopic imaging has been applied to map cerebral sO_2_ levels by collecting reflectance scattering light from brain tissue. However, this method is limited by insufficient spatial resolution, and microcirculation cannot be observed^42,43^. By using an oxygen-sensitive fluorescent probe, a two-photon phosphorescent lifetime microscope (2PLM) can be used to measure and image the partial pressure in vessels by recording the fluorescence decay time. However, accurate oxygenation quantification requires averaging multiple measurements, and this method is limited in terms of its temporal resolution^38,42,44^. In contrast, the proposed photoacoustic fiberscope is advantageous due to its light weight, high temporospatial resolution and oxygenation measurement capability. The fiberscope can be used to elucidate cerebral hemodynamics and oxygenation and can potentially be applied to detect cerebral dysfunction, study therapy responses, and monitor brain functional development. For example, cerebral vessels can dynamically regulate blood flow and oxygen supply differently in veins and arteries. The response magnitude and temporal process of Hb and sO_2_ changes are of great interest to understanding this response mechanism. Such changes can occur in seconds and can be measured at high temporal resolution by the photoacoustic fiberscope. Multi-modal or multi-contrast head-mounted microscopes have been developed for freely moving rodents to provide a comprehensive understanding of cerebral neurological and hemodynamic activities^45^. The photoacoustic fiberscope can be integrated with existing microscopes by utilizing the common optical path^46,47^. This is because the photoacoustic fiberscope does not require an optical/acoustic confocal scheme, and the small-sized sensor can be placed away from the laser beam. By using additional excitation wavelengths, we may be able to provide more molecular information, such as water and lipid distribution in the brain^48^.

The recently reported head-mounted photoacoustic microscopes detect laser-induced ultrasound waves using piezoelectrical transducers^17^. However, the tradeoff between the transducer size, detection sensitivity, and field of view hinders the oxygenation imaging capability of such devices. The proposed photoacoustic fiberscope uses a fiber optic sensor for high-sensitivity ultrasound detection, addressing this limitation. Compared with piezoelectric detection systems, optical ultrasound sensors can translate acoustic displacement into phase or intensity modulations based on the light signal, significantly amplifying the detection sensitivity^49^. Additionally, the proposed system uses a lightweight headpiece, reducing the requirements for precise optical and acoustic alignment. In our previous work, we developed a miniatured photoacoustic imaging probe that utilized this sensor for gastrointestinal endoscopy. We demonstrated its sO_2_ measurement capability but the probe required rotational scanning and took ~30 min to form an image^25^. In this work, the large-acceptance angle of the sensor was further exploited, enabling fast scanning imaging to capture dynamic changes in cerebral oxygenation at single-vessel resolution.

In our imaging results, we observed fluctuations in measured sO_2_ along individual vessels. These fluctuations can be attributed to optical scattering in biological tissue and absorption saturation effect. Randomized optical scattering can distort the laser light and lead to spatial inhomogeneity in the excitation light intensity. The absorption saturation effect can introduce additional errors in sO_2_ measurement due to the limited bandwidth of the transducer. This leads to nonlinear variations in the peak-to-peak amplitude of the photoacoustic signal with absorption coefficient. In addition, we used an optical-fiber slip ring to transmit the laser pulses from console to the imaging probe. However, the assessable slip ring is based on single mode fibers in communication band, and supports a few more modes at the visible band. The motion-induced bending and vibration of the fiber may cause a randomized fluctuation in the excitation pulse energy at the cortex. This issue may be addressed by optimizing the slip ring scheme which supports a single transverse optical mode.

The proposed fiberscope showed superior imaging capabilities; however, the current imaging instrumentation has some limitations. We aim to improve the imaging system in terms of the following aspects. First, the weight of the headpiece can be reduced by using polymer lenses instead of glass lenses, and the metal mounters can be replaced with lighter materials. The current headpiece is about 15% ~ 18% of the body weight of the experimental animals. Despite the minimal impact on the free moving capability, further reductions in the weight of the probe will be required in order to investigate more extensive and detailed activities. Second, the photoacoustic fiberscope cannot visualize vessels in deeper layers due to the limited imaging depth to about 160 µm by optical scattering. To overcome this limitation, near-infrared wavelengths can be utilized, which reduces the effects of tissue scattering and enable penetration depths on the order of millimeters^9,14^. Third, the field of view is limited by the maximum scanning angle of the MEMS mirror and the field of view of the object lens. The current imaging system uses achromatic doublets with an aperture of only a 1.4 mm. A compound lens with a flat field and low aberration could be introduced to extend the field of view. Fourth, to enhance axial resolution and improve the accuracy of sO_2_ measurement, we propose reducing the acoustic-impedance mismatch to increase the detection bandwidth of the sensor.

## Materials and methods

### Animal preparation

All animal procedures were conducted based on the “Guiding principles in the care and use of animals” (GB/T 35892-2018, China) and were approved by the laboratory animal ethics committee of Guangzhou Huateng Biomedical Technology (IACUC: HTSW220304). We fed C57BL/6 mice (4 weeks old, 15–25 g in weight) either a control diet (*n* = 10, five males and five females) or a high-fat diet (*n* = 30, fifteen males and fifteen females). The mice were housed in individual cages in an environment with a 12-h light-dark cycle at a temperature of 25 °C with food and water ad libitum. We measured the total cholesterol (TC), triglyceride (TG), high-density lipoprotein cholesterol (HDL-C), and low-density lipoprotein cholesterol (LDL-C) via blood tests with a lipid panel every 4 weeks (see Fig. S15). We measured the body weight every 2 weeks. After 8 weeks of feeding, the healthy and obese groups were used for cerebral imaging. The illumination fluence of the excitation laser in the cerebral tissue was in a safe range, following the American National Standard Institute (ANSI) safety standard.

### Hypercapnia experiment under anesthesia

We performed systemic hypoxia experiments with 1.5% isoflurane anesthesia. We placed the experimental animal on a heating pad to maintain a 37-degree body temperature. Hypercapnia was induced by changing the inspiratory air from normal to a 50:50 air/CO_2_ mixture. After 100 s of baseline imaging, we induced hypercapnia over 50 s and imaged the resultant changes in the cerebral vascular structure and blood sO_2_ levels.

### Hypercapnia experiment with freely moving mice

We performed hypercapnia experiments with freely moving mice in a gas chamber with a diameter of 40 cm and a height of 30 cm. The chamber has a gas input and an output at the bottom. After 100 s of baseline imaging, we pumped the air/CO_2_ mixture at 20 L/min to induce hypercapnia through the inlet. Then, normal air was pumped at 10 L/min to replace the air/CO_2_ mixture to recover to normal conditions. Standard atmospheric pressure was maintained in the tank.

### Preparation for cerebral imaging

We created a transcranial window under 1.5% isoflurane anesthesia. The mouse head was fixed in a stereotaxic apparatus with a head fixture device and positioner for precise positioning. First, the scalp was shaved with an electric razor, and a 1.5 cm incision was made along the middle line. Then, the scalp was removed to expose the whole skull, and the periosteum on the skull was removed using a scalpel and scissors. The skull of the region of interest (ROI) was then partially removed using a surgical dental drill, during which saline flushing was performed periodically to prevent overheating and bleeding. After window preparation, saline was used as a medium between the imaging probe and the mouse head.

For freely moving state imaging, we performed the following additional steps. First, the skin incision was treated with a tissue adhesive (3 M Vetbond) after the surface of the skull was dried. Then, the ROI was identified, and a customized holder was fixed to the skull with cyanoacrylate (Loctite 454). Afterward, dental adhesive resin cement (Sun Medical, Super-Bond C&B) was deployed to fill the remaining exposed skull and fix the holder. Finally, ultrasound gel was used to fill the gap between the tissue surface and the sensor. The head-mounted imaging device was fixed to the holder via magnetic force using four magnets. We adjusted the focal length of the laser beam before imaging to optimize the imaging performance (see Fig. S1 and Fig. S2 for details).

### Tracking the motion trajectory of the mouse

We recorded the motion trajectories of the freely moving mice with a video camera. The camera was placed on the top of the chamber, and a wide-angle lens was used to cover the entire motion area of the mouse. The chamber floor has a significant contrast in color to the mouse, and the animals were located via computer vision software. The 1920 × 1080 pixel videos were recorded at 30 frames per second. We used an OpenCV-based channel and spatial reliability tracking (CSRT) tracker to plot the motion trajectory. We selected the mouse head and the head-mounted microscope as the tracking targets. In the first frame, to initialize the tracker, the algorithm reads and learns the geometric and colorimetric features of the selected target. In the following frames, the target is found based on the pretrained features, and its position in (x, y) coordinates is recorded. Finally, the recorded coordinates in each frame are read out to plot the motion trajectory.

### Imaging data analysis and statistics

Figure S7 shows the flowchart showing the extraction process of arteriovenous Hb, sO_2_ and vessel diameters. The Hb photoacoustic images were generated by capturing the amplitude of the 532-nm excited photoacoustic signal (PA_532_) at each pixel using the maximum-amplitude projection method. Subsequently, sO_2_ values for each pixel were calculated via the spectral unmixing by using the measured amplitudes excited at 532 nm (PA_532_) and 558 (PA_558_) nm, based on the absorption spectra of the oxy- and deoxygenated hemoglobin, expressed as

1 $${\rm{s}}{{\rm{O}}}_{2}=\frac{\frac{{{PA}}_{558}}{{{PA}}_{532}}{\mu }_{532}^{{\rm{Hb}}}-{\mu }_{558}^{{\rm{Hb}}}}{\frac{{{PA}}_{558}}{{{PA}}_{532}}\left({\mu }_{532}^{{\rm{Hb}}}-{\mu }_{532}^{{\rm{Hb}}{{\rm{O}}}_{2}}\right)-\left({\mu }_{558}^{{\rm{Hb}}}-{\mu }_{558}^{{\rm{Hb}}{{\rm{O}}}_{2}}\right)}$$

where ${\mu }_{532}^{{\rm{Hb}}}=40584$, ${\mu }_{558}^{{\rm{Hb}}}=54164$, ${\mu }_{532}^{{\rm{Hb}}{{\rm{O}}}_{2}}=43876$, and ${\mu }_{558}^{{\rm{Hb}}{{\rm{O}}}_{2}}=33456$ (cm^-1^∙mole^-1^) are the absorption coefficients.

For the statistical analysis, we created a vessel segmentation algorithm to identify the arteries and veins in each frame. First, the arteries and veins were manually identified in the first frame based on the sO_2_ level. This led to the creation of separate images for arteries and veins, from which arterial and venous masks were obtained through binarization. Here, arteries and veins with diameters <40 µm are also removed by using the mask filtering in the subsequent frames. Then, the average sO_2_ and relative Hb levels were calculated based on the mean value of the filtered results. After performing a 10-fold interpolation based on the images, we conducted Gaussian fitting in the direction perpendicular to the blood vessels. Next, we quantified the full width at half maximum (FWHM) as the local vessel diameter. A matching algorithm based on normalized cross-correlation (NCC) was used to correct the mask for each frame to compensate for the possible deformation or offset of the target vessels.

The OEF was calculated based on the ratio of the oxygen saturation difference between the arteries and veins: $\frac{{s}_{a}{O}_{2}-{s}_{v}{O}_{2}}{{s}_{a}{O}_{2}}$. From each imaging frame, we extracted the acquired hemodynamic parameters, and their variations are plotted in Figs. 2–5. The data presented in these figures were obtained from multiple mice that were subjected to CO_2_ or N_2_ respiration after the acquisition of baseline data. The temporal data between individual mice were synchronized based on the commencement of the gas injection. The synchronized time trace data are represented as the average (solid line) and standard error of mean (shaded error bar). The error bar indicates inter-mouse variability. We calculated the fractional changes of the recorded relative Hb and diameter variations with respect to baseline values, as shown in Figs. 2–5. We used two-way ANOVA to determine significance, and all statistical data are presented with the s.e.m., with *P* < 0.05 considered significant.

### Dual-wavelength pulsed laser source

We built a dual-wavelength pulsed laser source for multispectral photoacoustic imaging. The 532-nm laser light was emitted from a nanosecond laser (VPFL-G-HE-30, Spectra-Physics), and the 558-nm laser beam was obtained by optically pumping a single-mode fiber (HB450-SC, Fibercore) with another 532-nm nanosecond laser. The stimulated Raman scattering (SRS) effect induces energy conversion for the first-order Stokes wave at 545 nm and the second-order wave at 558 nm. We used a bandpass filter (ZET561/10x, Chroma) to filter out the 558-nm component. The first half-wave plate (GCL-060411, Daheng Optics) was used to adjust the light intensity of the Raman pump path with a polarizing beam splitter cube (PBS101, Thorlabs). We coupled the pump laser beams into the optical fiber through a collimator (F220FC-532, Thorlabs). We employed a second half-wave plate to adjust the laser polarization state to achieve a maximal efficiency >30% in the 532–558 nm wavelength conversion. The maximal pulse energy at 558 nm can reach 2.0 μJ. Two neutral density filters (NDC-50C-4 M, Thorlabs) were used to adjust the intensities of the 532-nm and 558-nm components. A dichroic mirror (ET550lp, Chroma) was applied to combine the two-color laser beams. The laser beam was coupled into a single-mode fiber and delivered to the imaging probe. The single pulse energy was 200 nJ.

### Ultrasound detection with the fiber optic sensor

We used a fiber optic sensor to detect laser-induced ultrasound waves. We ultraviolet-inscribed two highly reflective index gratings (4 mm each in length and 3 mm in separation) to form a high-finesse cavity in the erbium/ytterbium codoped core of an optical fiber (EY305, CorActive). This sensor works in laser mode, unlike the passive optical resonators used in previous works. The rare-earth dopant offers optical gain, and the two reflectors provide optical feedback. A 980-nm low-coherence semiconductor laser pumped the cavity to lase at a single longitudinal mode at 1530 nm with a kilohertz linewidth. The incident ultrasound wave can deform the sensor and induce a lasing-frequency variation.

The acoustically induced laser frequency change is read out via heterodyne optical detection^49^. The sensor laser naturally has two orthogonally polarized modes with slightly different lasing frequencies. The two laser beams pass through a 45-degree polarizer and generate a beat signal at the high-speed photodetector. Their beat signal falls in the radio frequency range (~2 GHz). The ultrasound wave-induced identical frequency variations to the *x*- and *y*-polarized laser beams have opposite signs. As a result, the acoustically induced lasing frequency change can be measured in the radio frequency range.

We used a multifunction reconfigurable I/O module (NI PXIe-7852R) to generate a synchronized trigger signal. In addition, a vector signal transceiver (NI PXIe-5646R) was used to record the waveform of the frequency-downshifted signal generated by mixing the beat signal with a local reference microwave source. The phase variation and the frequency shift were acquired via I/Q demodulation. The sampling rate was 125 MHz, which allows an acquisition bandwidth of 62.5 MHz. In addition, we used the integrated FPGA in the transceiver to calculate the sO_2_ level and display the hemoglobin and sO_2_ images in real time. All the image sequences and pulse energies of the 558-nm laser component were saved in a high-speed disk array.

### Blood gas analysis

To perform a blood gas analysis with a mouse, we anesthetized the experimental animal with 1.5% isoflurane, obtained a small blood sample from the mouse by using a cardiac puncture technique, and then analyze the blood sample using a blood gas analyzer.

### Supplementary information

Supplementary Information

Supplementary videos

## Data Availability

The data that support the findings of this study are available from the authors upon reasonable request.

## Code availability

The authors declare that for data collection the commercially available software from LabVIEW 2015 (National Instruments, USA) and Matlab 2018b (Matlab, Mathworks, USA) were used. Data analysis was conducted in Matlab using its built-in functions. The image algorithm has been patented and is available upon discretion from the corresponding author.
