# Supplementary material for: Free-moving-state microscopic imaging of cerebral oxygenation and hemodynamics with a photoacoustic fiberscope
Source: Light Sci Appl. 2024 Jan 2;13:5. doi: 10.1038/s41377-023-01348-3 (PMC10758391; doi:10.1038/s41377-023-01348-3)
Supplement: Supplementary file 1 — Supplementary Information [file 41377_2023_1348_MOESM1_ESM.docx]

**Supplementary information for**

**Free-moving-state microscopic imaging of cerebral oxygenation and hemodynamics with a photoacoustic fiberscope**

Xiaoxuan Zhong^1,2^, Yizhi Liang^1,2^, Xiaoyu Wang^1^, Haoying Lan^1^, Xue Bai^1^,

Long Jin^1,*^ and Bai-Ou Guan^1,*^

^1^*Guangdong Provincial Key Laboratory of Optical Fiber Sensing and Communications, Institute of Photonics Technology, Jinan University, Guangzhou 510632, China.*

^2^*These authors contributed equally: Xiaoxuan Zhong, Yizhi Liang*

^*^*Corresponding author: tjinlong@jnu.edu.cn; tguanbo@jnu.edu.cn*

Figure S1. Headpiece assembly.

Figure S2. Procedure for installing the headpiece.

Figure S3. MEMS driving and sensor interrogation unit.

Figure S4. Dual-colored laser source.

Figure S5. Spatial resolution.

Figure S6. Depth calibration.

Figure S7. A flow chart outlining the extraction process of arteriovenous Hb, sO_2_, and blood vessel width.

Figure S8. Observation of cerebrovascular responses to 50% CO_2_ respiration under anesthesia using a stereomicroscope and a photoacoustic fiberscope.

Figure S9. Statistics for the cerebrovascular responses in 50% CO_2_ respiration experiments under anesthesia.

Figure S10. Observation of cerebrovascular responses to 10% CO_2_ respiration under anesthesia.

Figure S11. Cerebrovascular responses to 10% N_2_ respiration under anesthesia.

Figure S12. Statistics for the cerebrovascular responses in the N_2_ hypoxia experiments under anesthesia.

Figure S13. The behavior of freely moving mice with and without mounting the photoacoustic fiberscope.

Figure S14. Behavior of freely moving healthy and obese mice with a head-mounted fiberscope during 50% CO_2_ respiration.

Figure S15. Physiological indices of healthy and obese mice.


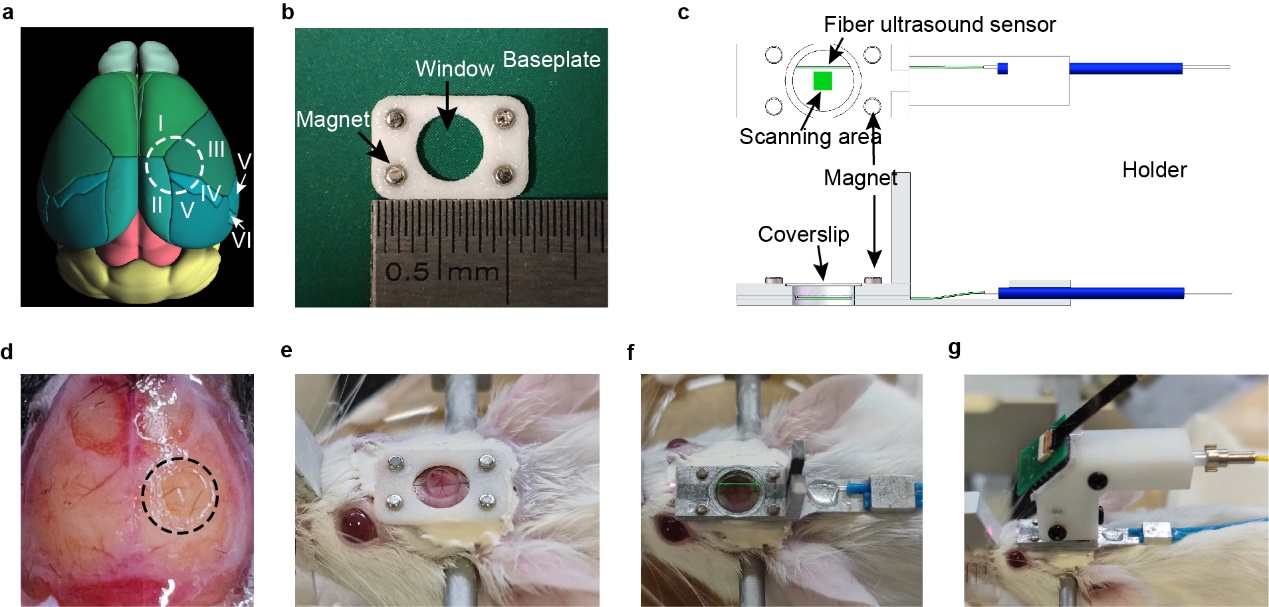


**Figure S1. Headpiece assembly**. (a) Mouse brain atlas. I: Somatomotor areas; II: Retrosplenial areas; III: Somatosensory areas; IV: Posterior parietal association areas; V: Visual areas; VI: Auditory areas; VII: Temporal association areas^1^. The white dashed circle indicates the location of the cranial window. (b) Photograph of the baseplate. (c) Schematic of the sensor holder. (d) Photograph of the cranial window. The black dashed circle indicates the location of the cranial window. (e) Baseplate mounted over the cranial window. (f) Ultrasound sensor and its holder mounted on the head. (g) The headpiece assembly was completed by screw-fixing the laser scanning unit onto the sensor holder.


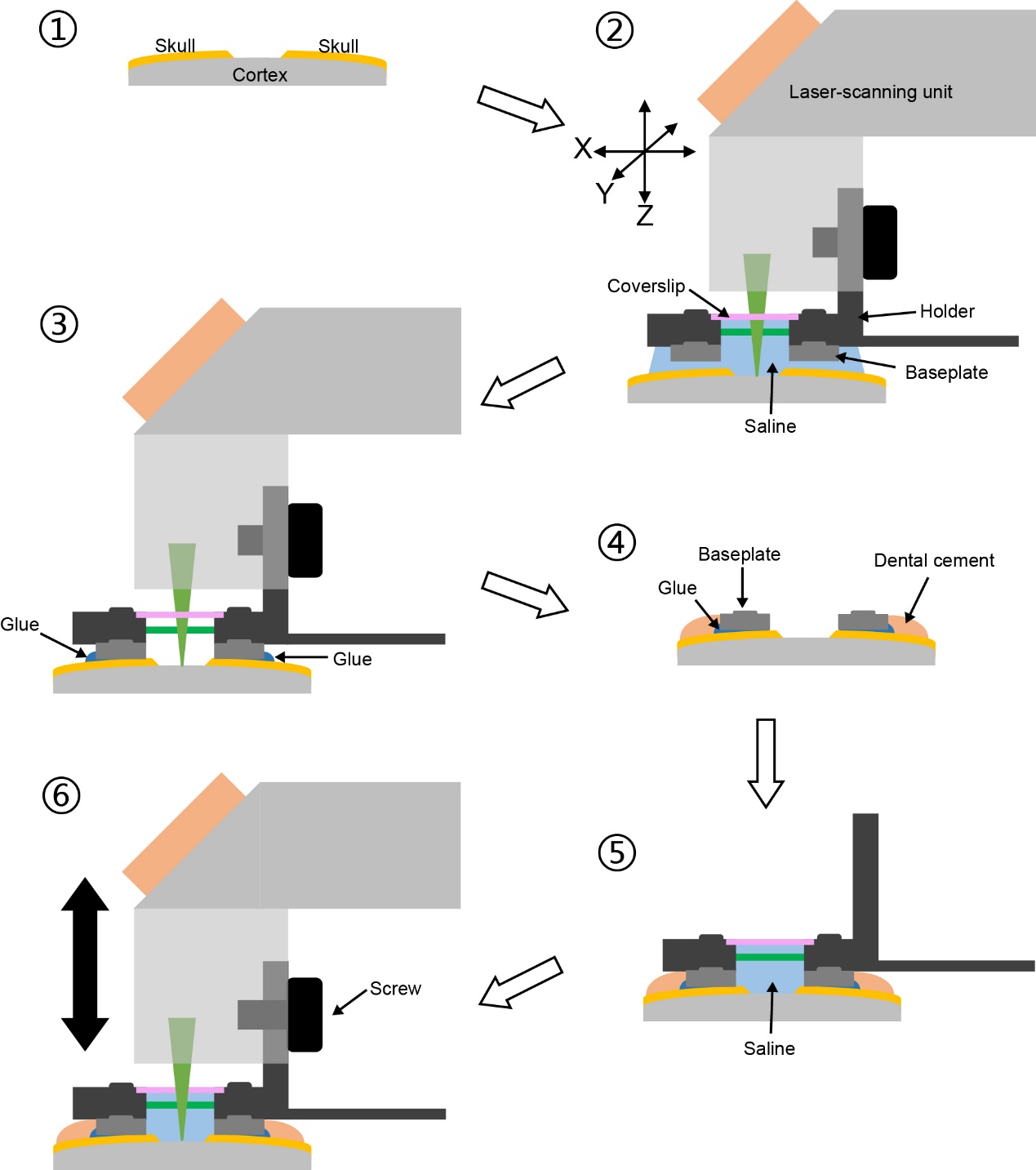


**Figure S2. Procedure for installing the headpiece.** Step 1: Create a cranial window. Step 2: Identify the region of interest (ROI) during continuous imaging while scanning the imaging probe with a 3-axial motorized stage. In this step, an acoustic coupling medium (saline) is used for ultrasound detection. Step 3: Attach the baseplate and sensor holder to the skull. Step 4: Remove the sensor holder from the baseplate and use dental cement to stabilize the baseplate. Step 5: Fill the ROI with a saline drop and reattach the sensor holder to the baseplate. Step 6: Adjust the focus before fixing the laser-scanning unit on the sensor holder.


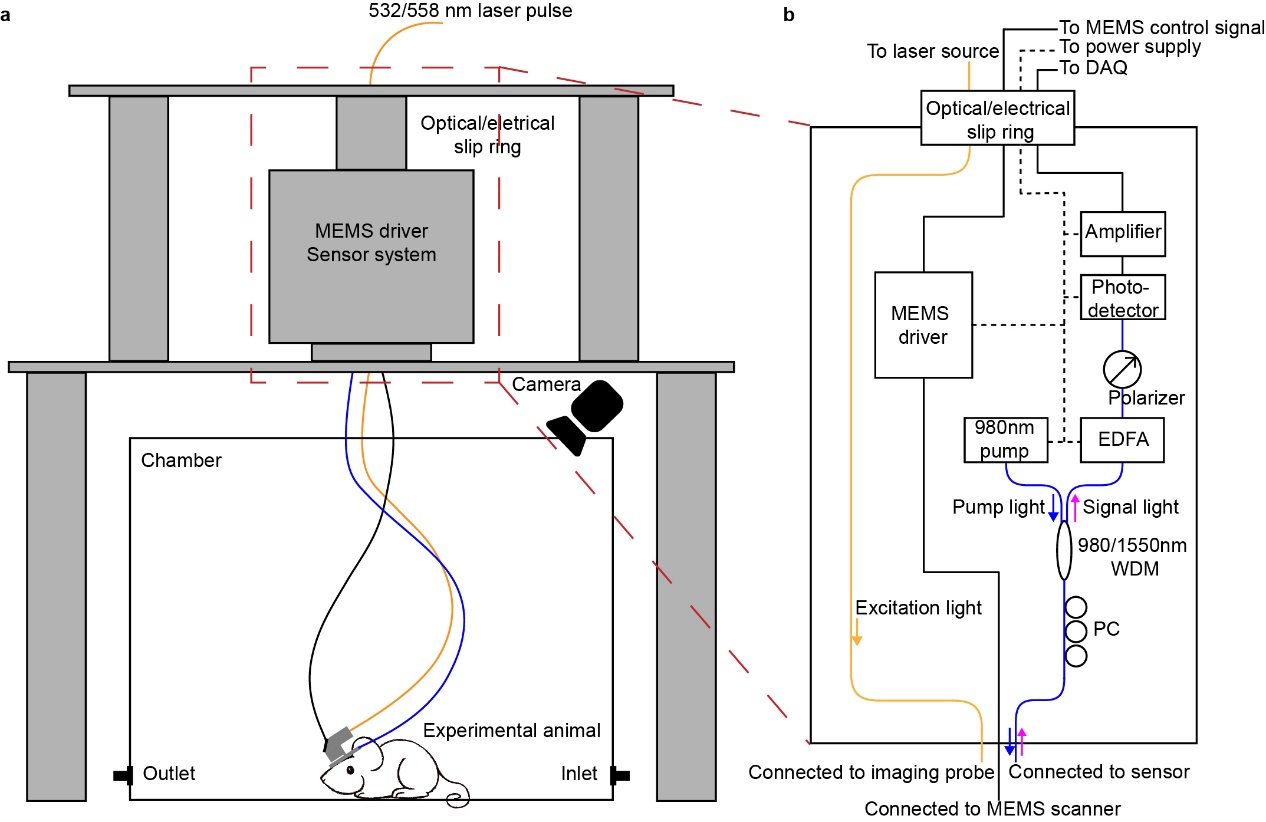


**Figure S3. MEMS driving and sensor interrogation unit.** DAQ, data acquisition; MEMS, microelectromechanical system; EDFA, erbium-doped fiber amplifier; PC, polarization controller.


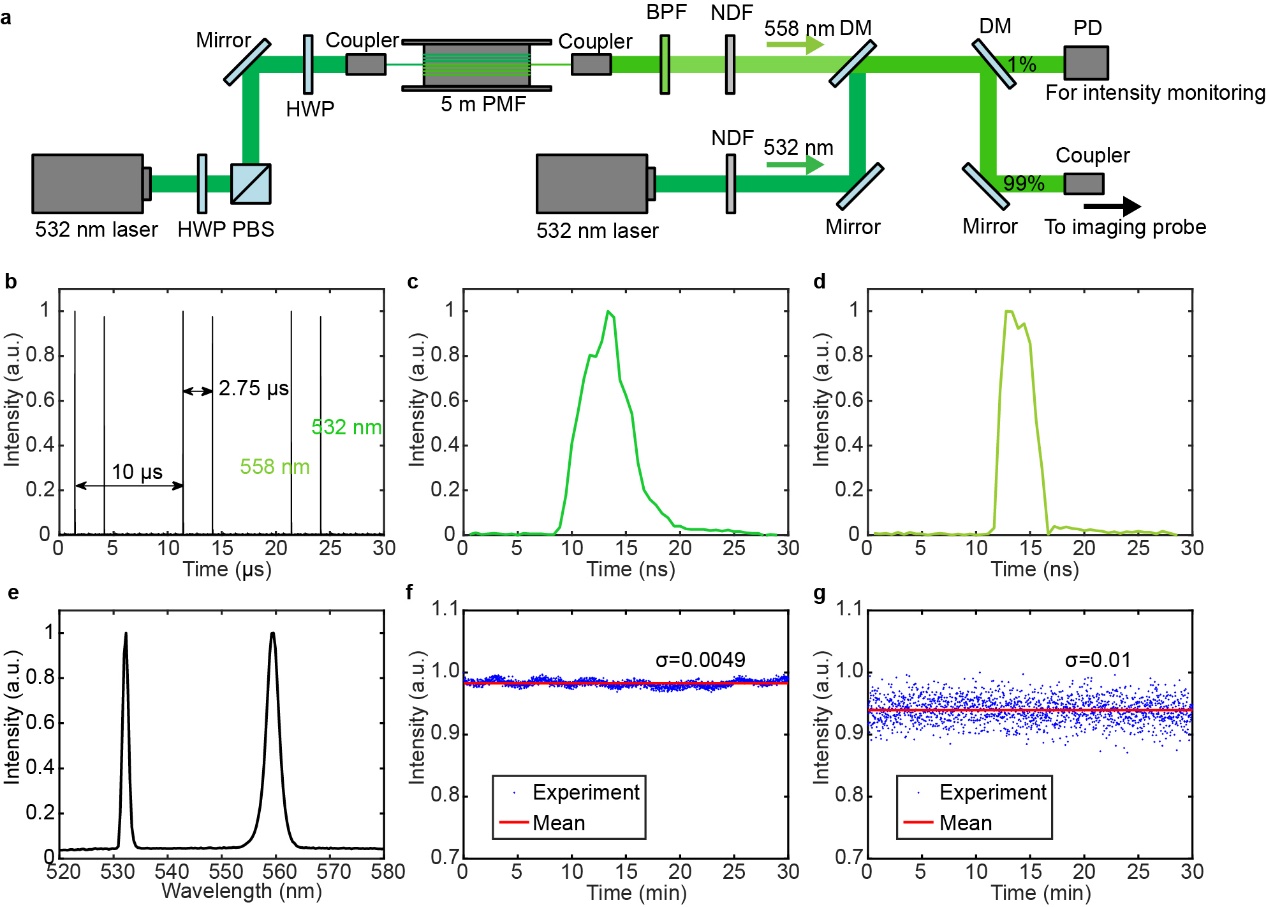


**Figure S4. Dual-colored laser source**. (a) Schematic of the laser source. HWP, half-wave plate; PBS, polarization beam splitter; PMF, polarization-maintaining fiber; BPF, bandpass filter; NDF, neutral density filter; DM, dichroic mirror; PD, photodetector. (b) Temporal profile of the laser output. (c) The measured waveform of a 532 nm laser pulse. (d) The measured waveform of a 558 nm laser pulse. (e) Optical spectrum of the laser source. Output power stability test results for 532 nm (f) and 558 nm (g) laser sources over a 30-minute period, with $\sigma$ standing for standard deviation.


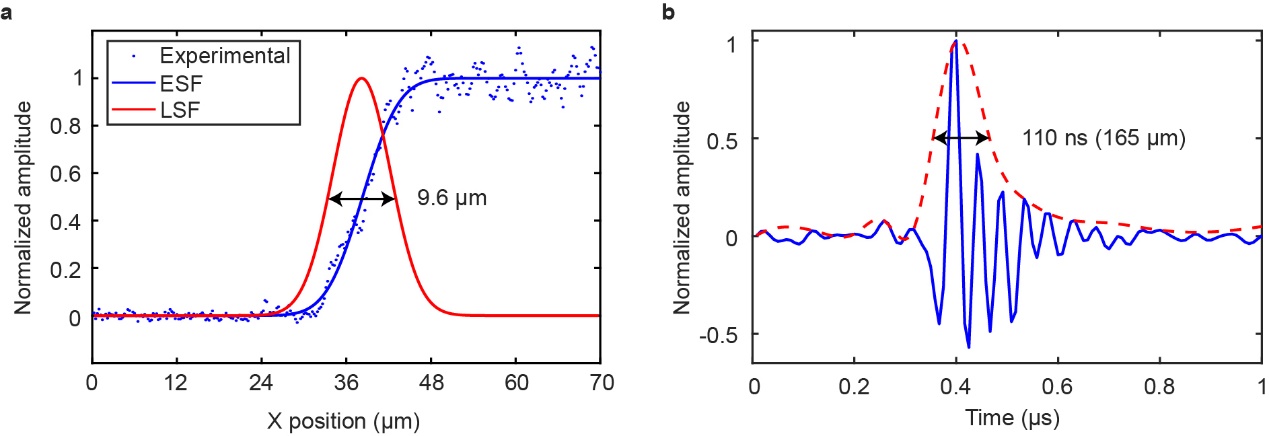


**Figure S5. Spatial resolution**. (a) Lateral. (b) Axial. The lateral resolution of the imaging system was evaluated by imaging the edge of a sharp blade. The edge spread function (ESF) was fitted using the maximum amplitude projection (MAP) data across a B-scan. The first derivative of the ESF was obtained to create a line spread function (LSF). The full width at half maximum (FWHM) of the LSF was considered as the lateral resolution, which was found to be 9.6 μm. To quantify the axial resolution, a highly absorptive black tape was imaged by using the photoacoustic fiberscope. Figure S5b shows the photoacoustic signal waveform detected by the fiber sensor. The axial resolution was determined by the full width at half amplitude of the envelope of the waveform. With a sound speed of 1500 m s^-1^, the axial resolution was estimated to be 165 μm. The axial resolution is determined by the detection bandwidth of the ultrasound sensor, which was limited by the mismatch between fiber glass and water in acoustic impedance.


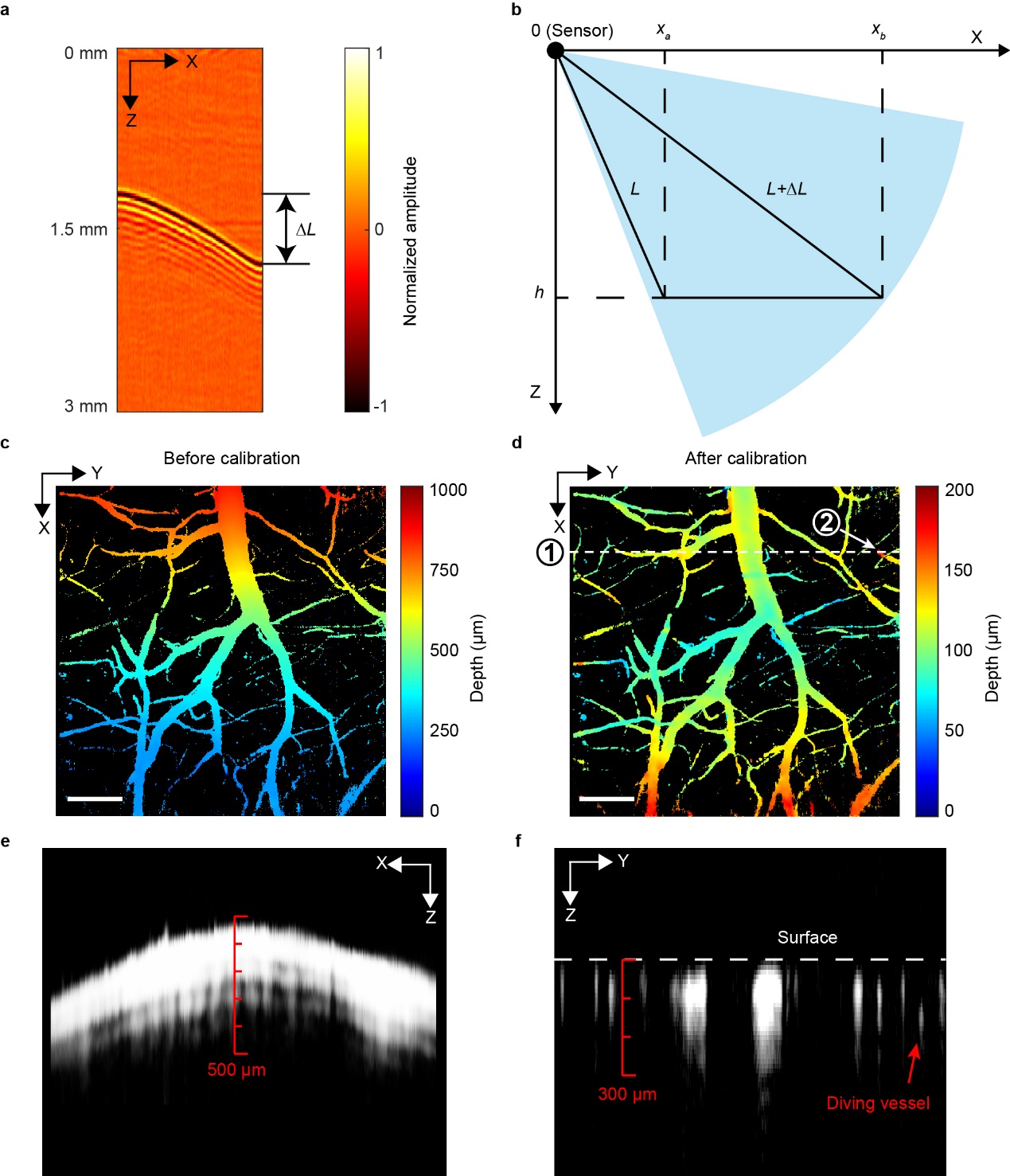


**Figure S6. Depth calibration.** (a) B-scan image of a flat black tape before depth calibration. Here, the curved image is a result of the varying time-of-flight at each laser-scanning step. (b) Geometric relation between the sensor and the imaging area, where *h* represents the distance between the sensor and the imaging plane, *x*_a_ and *x*_b_ define the imaged range. The blue shaded region indicates the acceptance angle of the sensor. In this context, ∆*L* represents the distance difference from the sensor to the starting point *x*_a_ and finishing point *x*_b_, where *L*^2^=(*x*_a_^2^+*h*^2^) and (*L*+∆*L*)^2^= *L*^2^=(*x*_b_^2^+*h*^2^). We conducted a depth calibration based on the geometric relations illustrated in Figure b, with measured value of ∆*L*. (c) and (d) Imaging results of cortical vessels before and after depth calibration. (e) Maximum amplitude projection image at the coronal plane (*x*-*z* plane) after calibration. (f) B-scan image at the dashed line (① in Figure S6d) after depth calibration. A diving vessel is marked as ② in Figure S6d. Scale bar, 200 μm.


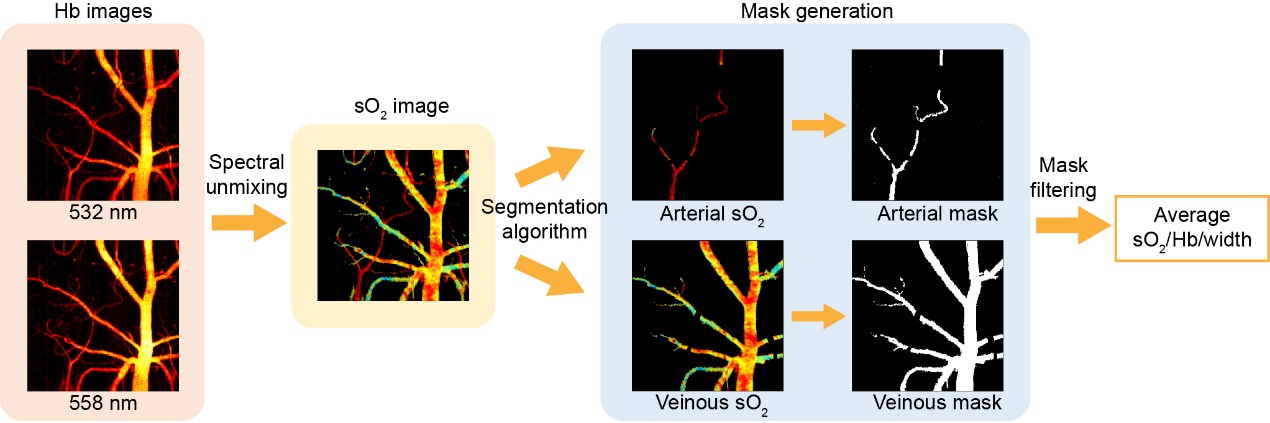


**Figure S7. A flow chart outlining the extraction process of arteriovenous Hb, sO_2_, and blood vessel width.** This extraction process was performed for each frame, resulting in time-traced variations that are shown in Figure 2, Figure 4, and Figure 5 in the main text, as well as Figure S10 and Figure S11.


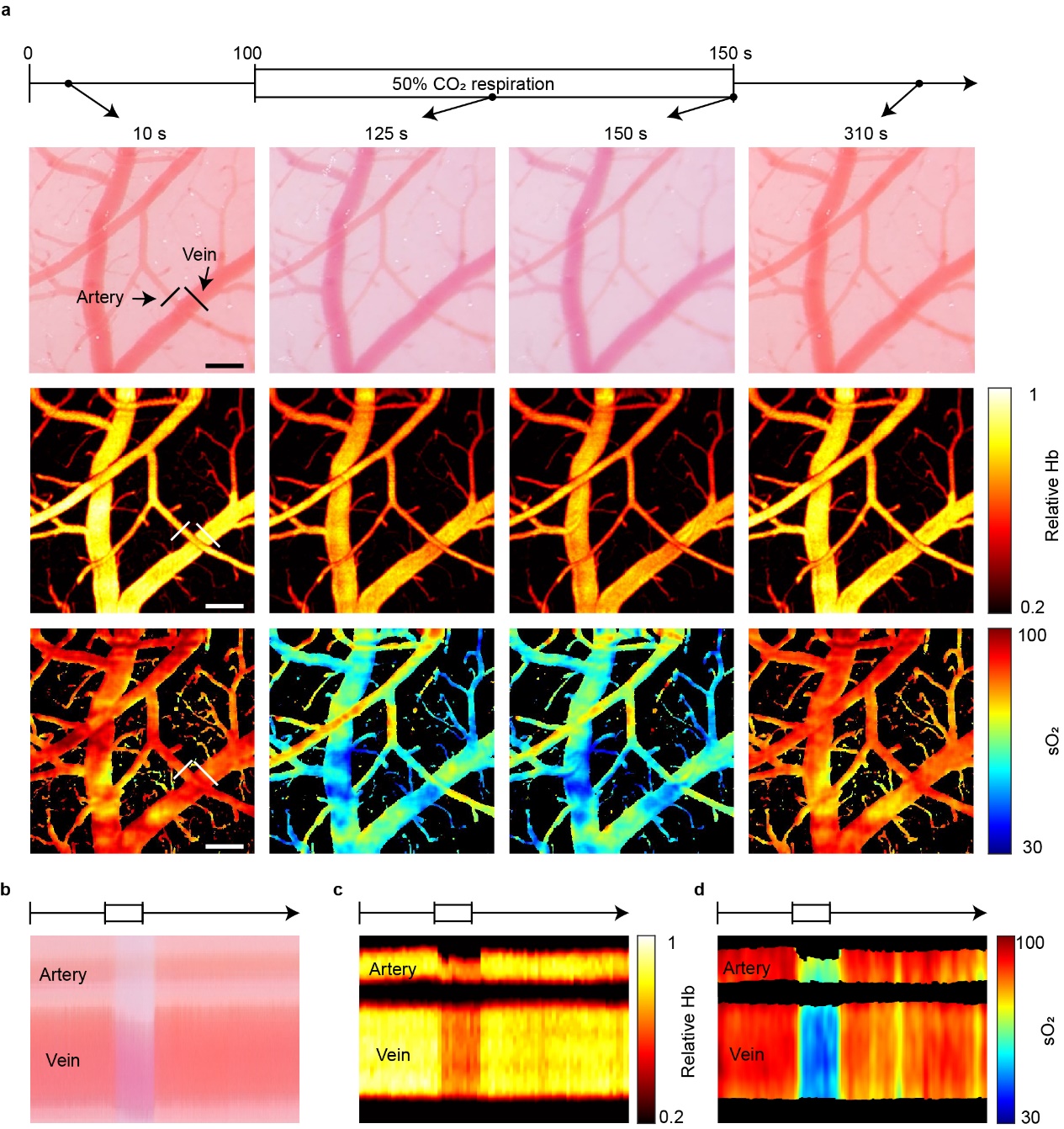


**Figure S8. Observation of cerebrovascular responses to 50% CO_2_ respiration under anesthesia using a stereomicroscope and a photoacoustic fiberscope.** (a) Snapshot images captured by the stereomicroscope (top panel), Hb (middle panel) and sO_2_ (below panel) photoacoustic images during the high-concentration CO_2_ respiration. (b-d) Kymographs of the selected artery and vein indicated in Figure S8a imaged by the stereomicroscope (b) and the fiberscope (c for Hb and d for sO_2_). Scale bar, 200 μm.

For comparison, we conducted an additional hypercapnia experiment with 50% CO_2_ respiration and imaged the cerebrovascular response in the same target cortex region using a stereomicroscope (LS745, Laite) and our photoacoustic fiberscope. The recorded changes in blood vessel diameter and hemoglobin concentration were found to be in good agreement between the stereomicroscopic and photoacoustic images. However, the contrast-to-noise ratio of the stereomicroscope is lower compared to photoacoustic microscopy, particularly for capillaries and blood vessels at low concentrations. Additionally, the stereomicroscope lacks the ability to image functional information.


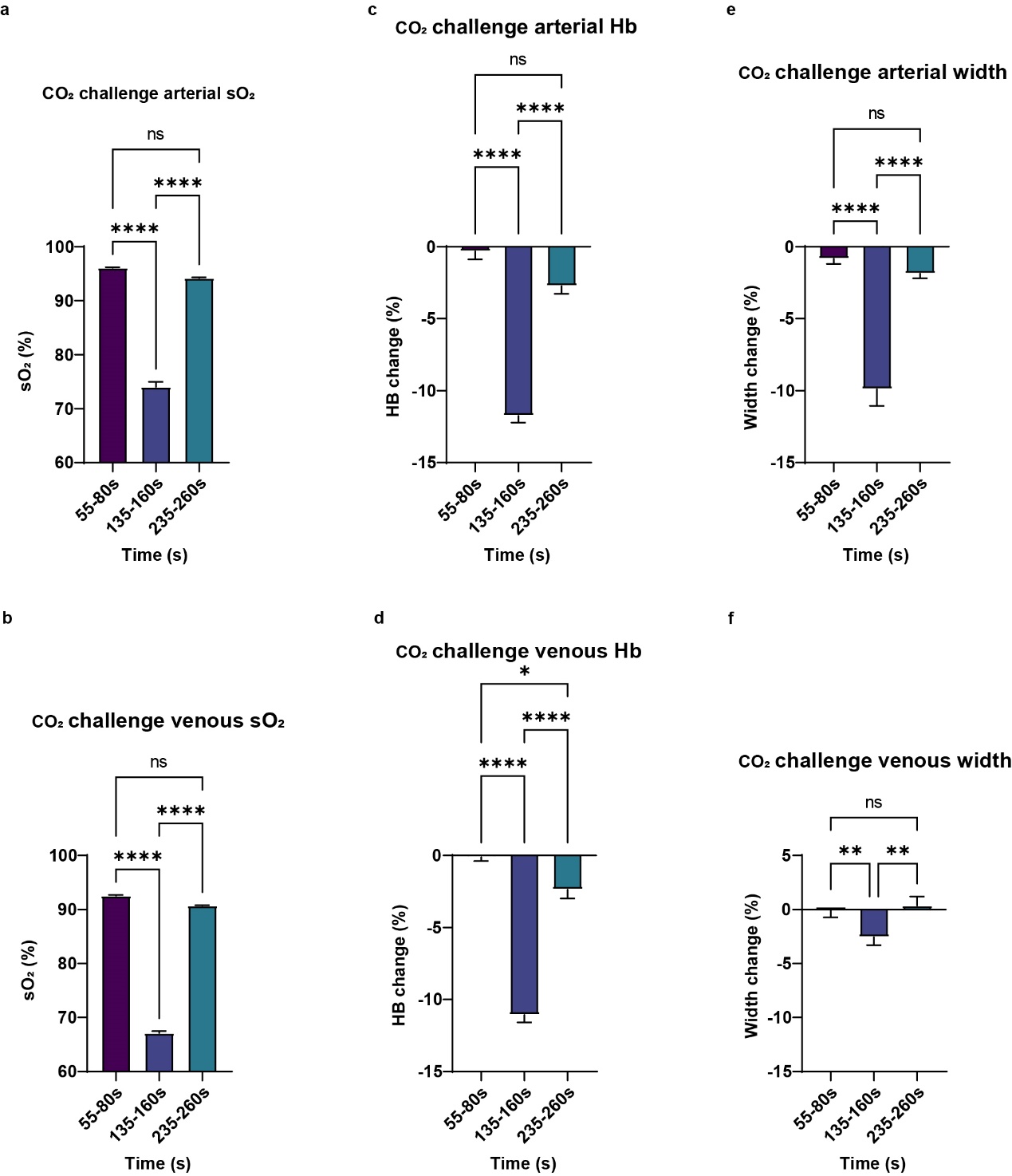


**Figure S9. Statistics for the cerebrovascular responses in 50% CO_2_ respiration experiments under anesthesia.** Statistical test results of the changes in the (a) artery sO_2_ level, (b) vein sO_2_ level, (c) arterial Hb level, (d) venous Hb level, (e) artery width, and (f) vein width during the four selected periods. The P values were determined by two-way ANOVA. * P<0.05, ** P<0.01, **** P<0.0001, ns not significant (n = 4).


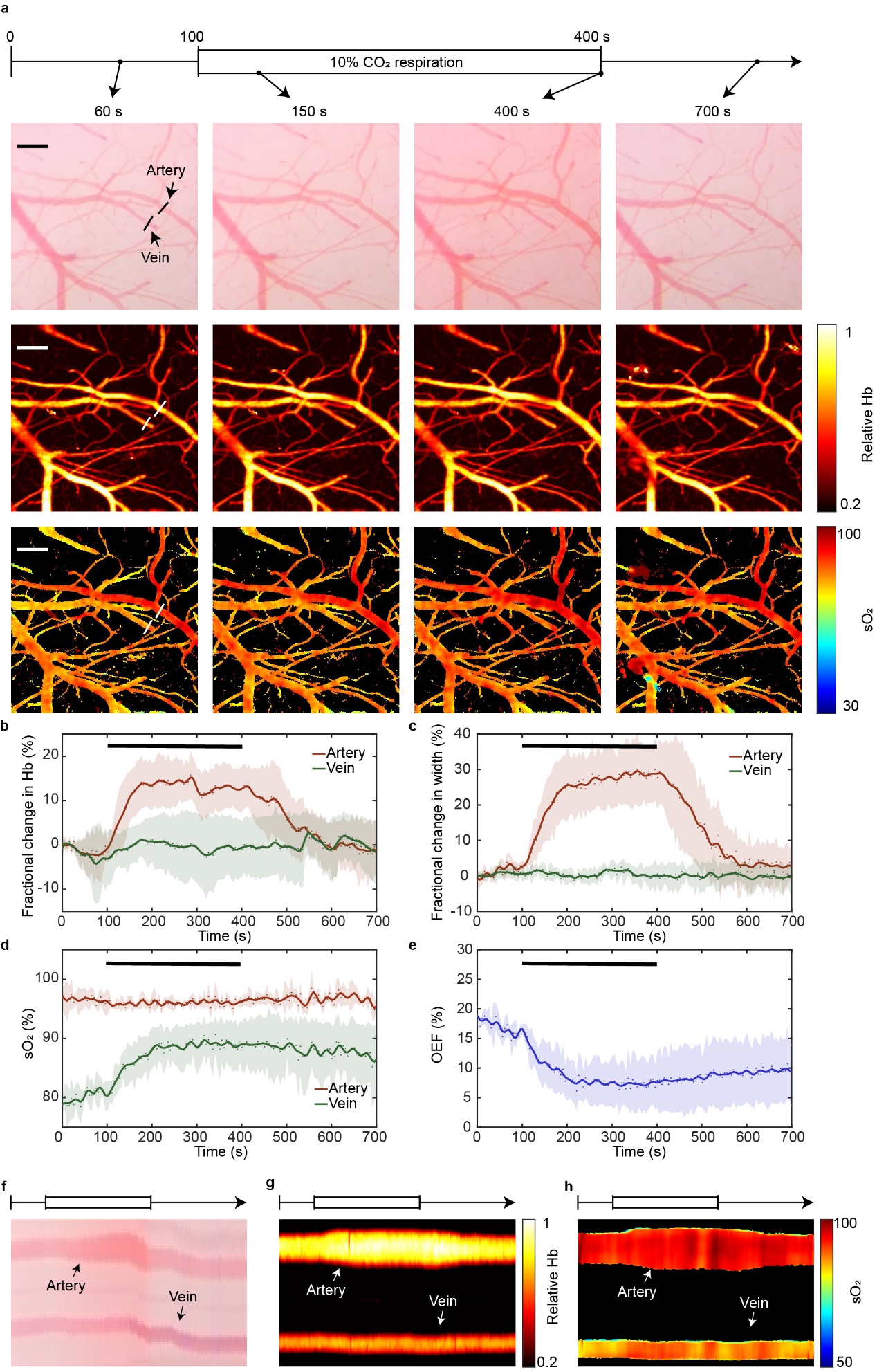


**Figure S10. Observation of cerebrovascular responses to** **10% CO_2_ respiration under anesthesia.** In (a), snapshot images of the stereomicroscope, Hb and sO_2_ photoacoustic images are presented. The recorded variations in Hb, vessel diameter, sO_2_, and OEF by the photoacoustic fiberscope are shown in (b-e), respectively. The data are presented as mean ± s.e.m., with n = 5. The black lines indicate the period of CO_2_ respiration over *t*=100 s to *t*=400 s. Kymographs of the selected vessels marked in Figure S10a before, during, and after the 10% CO_2_ respiration are shown in (f-h) for stereomicroscopy, Hb and sO_2_ photoacoustic fiberscope, respectively. Scale bar, 200 μm.

Notably, the stereomicroscope is not affixed to the mouse head, and the imaged blood vessels exhibit slow movement during imaging, as depicted in Figure S10f. Figure S10b shows an approximate 14% increase in arterial Hb levels, while Figure S10c exhibits a 25% vasodilation in an artery. Figure S10d demonstrates an increase in mean venous sO_2_ levels from 79% to 89%. The vessel diameter and Hb recovery process took nearly 200 seconds. Figure S10e shows a decrease in OEF from 18% to 7%. These results are consistent with previous studies. For instance, it has been reported that 10% CO_2_ respiration leads to a 15 to 30% change in arteriole diameter, while venous changes are less noticeable. Additionally, the increase in hemoglobin concentration and venous blood oxygen aligns with the previously measured blood oxygen increase reported in the literature^2-4^.


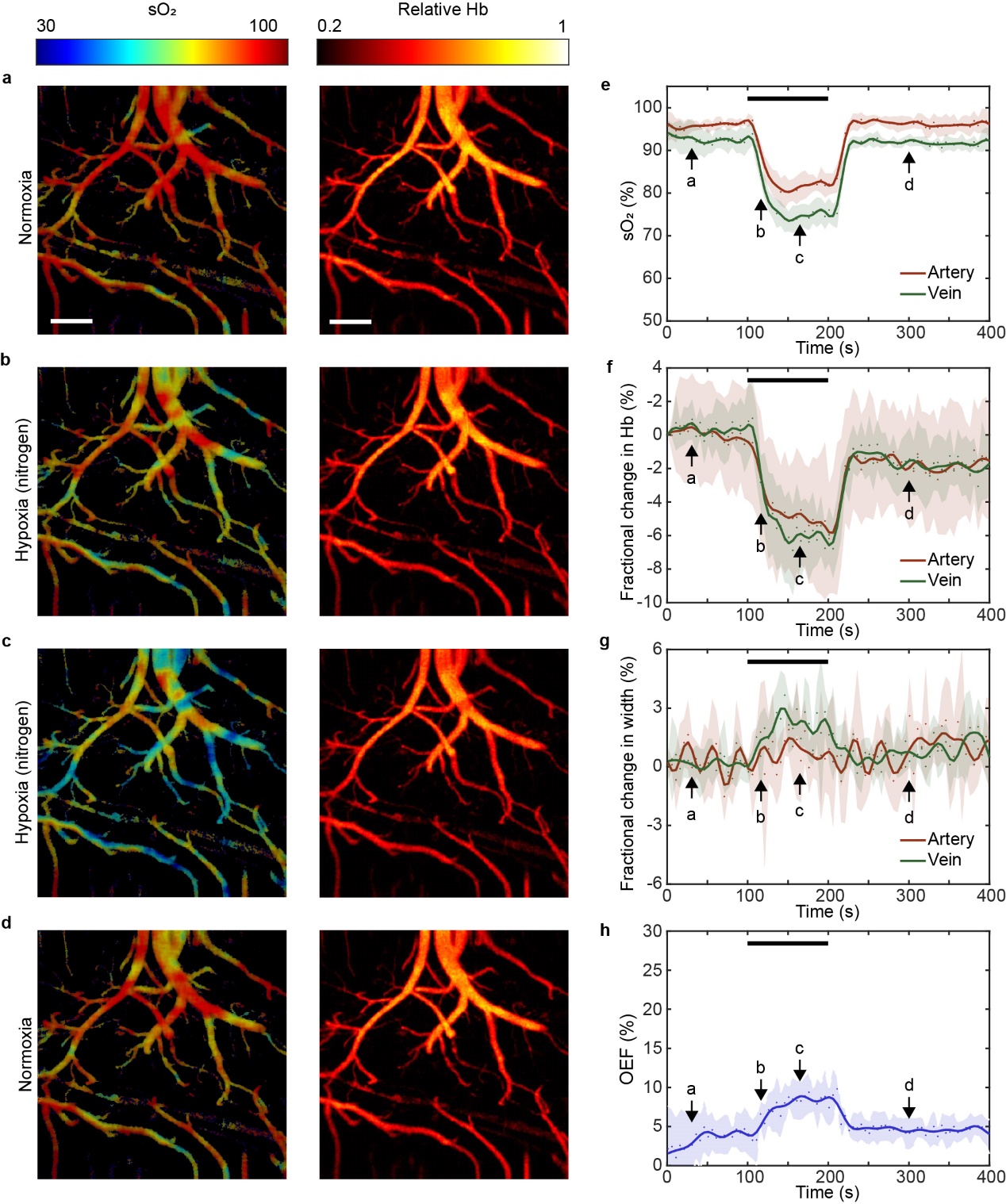


**Figure S11. Cerebrovascular responses to 10% N_2_ respiration under anesthesia.** (a-d) Selected snapshots of sO_2_ and Hb photoacoustic images in a normoxia-hypoxia cycle. (e-h) Variations in the sO_2_ level, Hb level, vessel width, and OEF during the hypoxia experiment. The black lines in (e-h) indicate the period with N_2_ respiration. n= 4; the data are shown as the mean ± s.e.m. Scale bar, 200 μm.


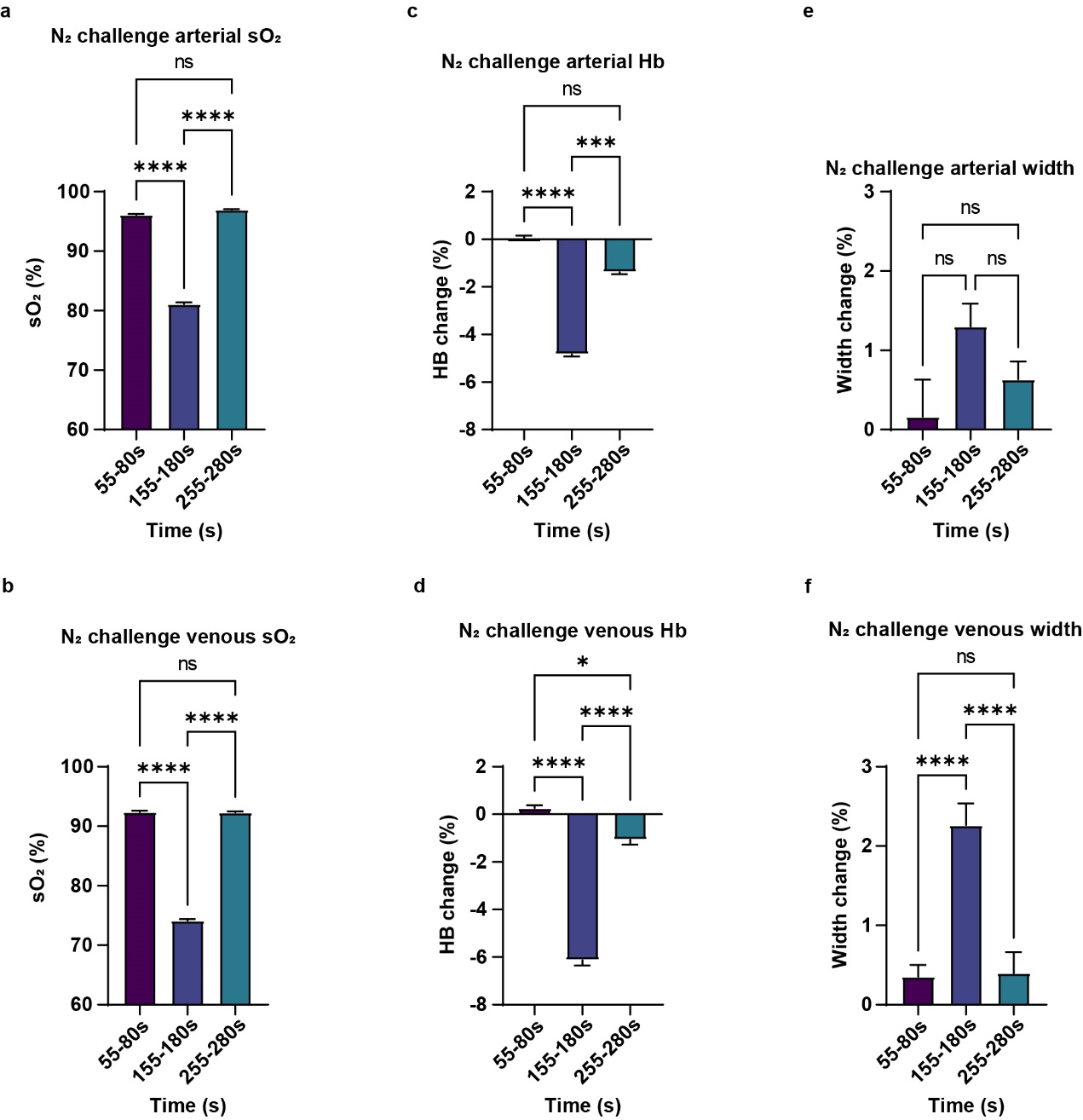


**Figure S12. Statistics for the cerebrovascular responses in the N_2_ hypoxia experiments under anesthesia.** Statistical test results of the changes in the (a) artery sO_2_ level, (b) vein sO_2_ level, (c) arterial Hb level, (d) venous Hb level, (e) artery width, and (f) vein width during the selected four periods. Here, the P values were determined by two-way ANOVA. *P<0.05, ***P<0.001, ****P<0.0001, ns not significant (n = 4).


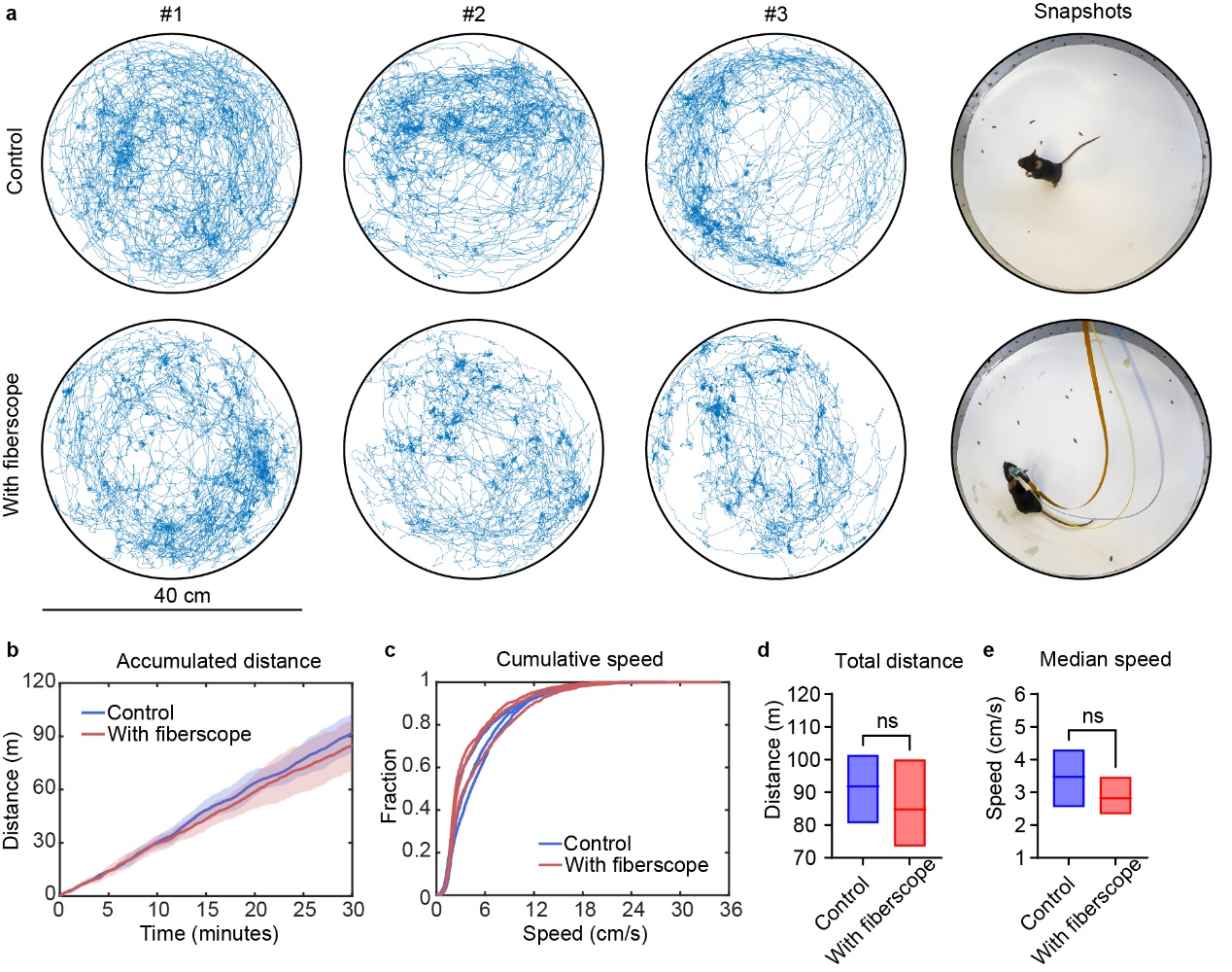


**Figure S13. The behavior of freely moving mice with and without mounting the photoacoustic fiberscope.** (a) Recorded trajectories of three mice (weights: 28 to 30 g) running in a 40 cm diameter area, with two trials per mouse: control (no fiberscope or cable, upper panel) and mounting a 4.5-g fiberscope with cable connections (lower panel). The snapshots captured by the camera are also provided. (b) Cumulative moving distance over 30 minutes, with curves representing the mean value across three mice and shaded regions indicating the s.e.m. at all-time points. (c) Cumulative speed distribution over 30 minutes of free motion, with each curve corresponding to one trial and color indicating control and with fiberscope, respectively. (d) and (e) The total moving distance and median moving speed with and without mounting the fiberscope, respectively. The data are presented as box plots from three mice with or without fiberscope, with horizontal lines indicating the mean and boxes representing the minimum and maximum range. A two-tailed paired t-test was conducted, with p-values for speed and distance being 0.3434 and 0.5208, respectively.


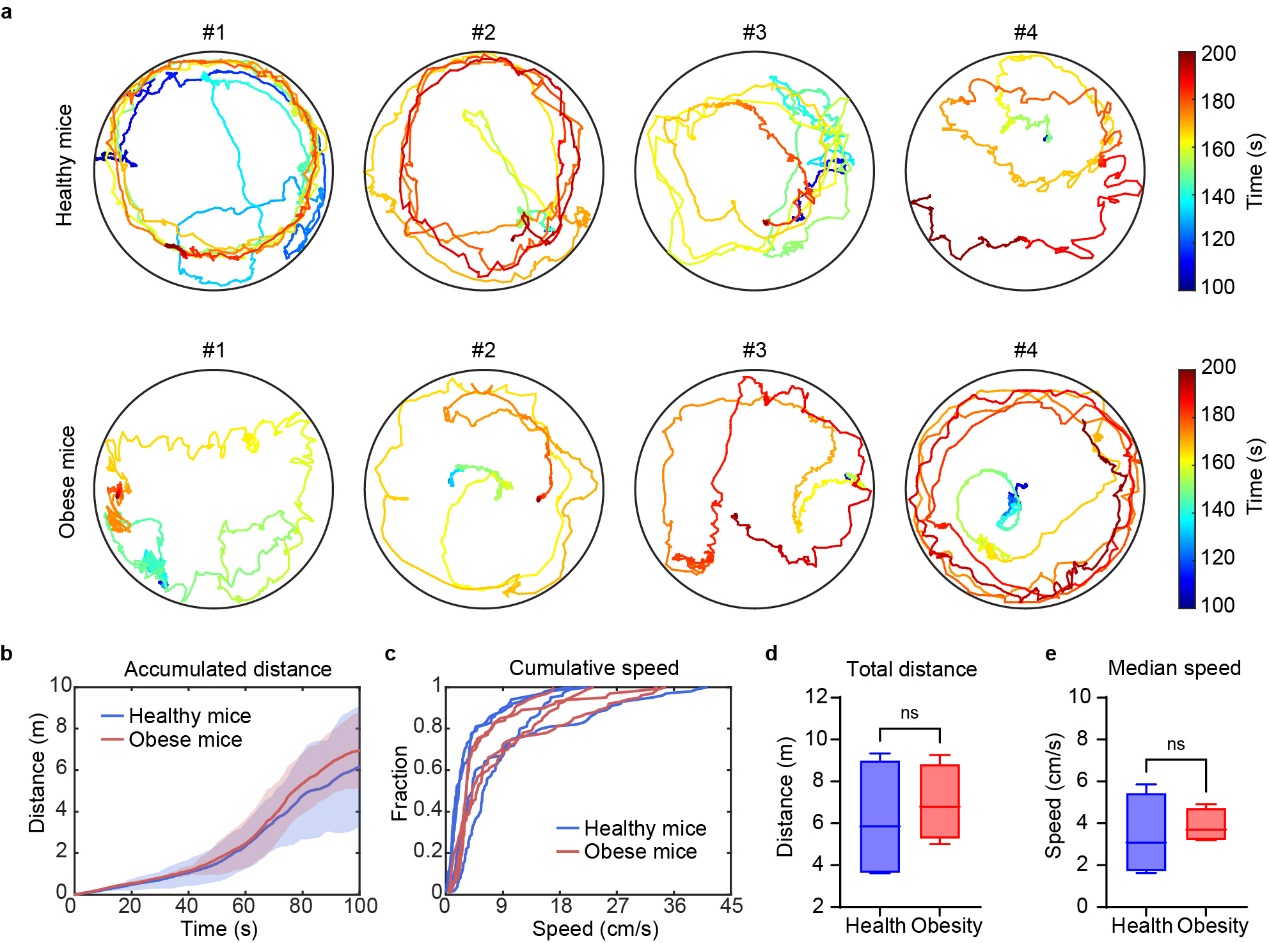


**Figure S14. Behavior of freely moving healthy and obese mice with a head-mounted fiberscope during 50% CO_2_ respiration.** (a) Recorded trajectories of four healthy mice (upper) and four obese mice (lower). (b) Cumulative moving distance over 100 seconds during CO_2_ respiration, with lines representing the mean across four mice and shaded regions indicating the s.e.m. at all-time points. (c) Cumulative speed distribution over 100 seconds during CO_2_ respiration, with each curve representing one mouse and color indicating healthy and obese mice, respectively. (d) and (e) Box plots for total moving distance and median moving speed, respectively, with horizontal lines indicating the mean, boxes representing the interquartile range, and whiskers representing the minimum and maximum range. A two-tailed paired t-test was conducted, with p-values for speed and distance being 0.6811 and 0.6619, respectively.


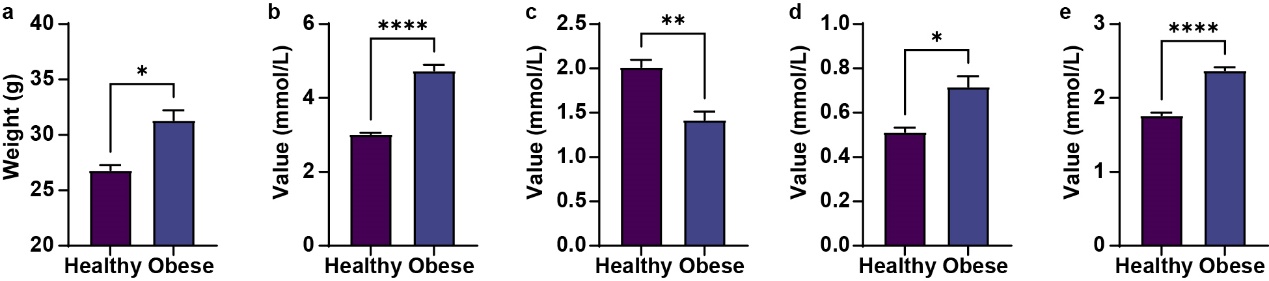


**Figure S15. Physiological indices of healthy and obese mice**. (a) Weight, (b) TC, (c) TG, (d) LDL-C, and (e) HDL-C of the mice after eight weeks of a high-fat diet. TC, total cholesterol; TG, triglyceride; HDL-C, high-density lipoprotein cholesterol; LDL-C, low-density lipoprotein cholesterol. P values were determined by t tests. * P<0.05, ** P<0.01, **** P<0.0001 (healthy mice n=5, obese mice n = 15).

**Supplementary Video Captions**

**Supplementary Video 1. Cerebrovascular response to hypercapnia.**

Hb (Right) and sO_2_ (Left) photoacoustic imaging result of animal under anesthesia, showing changes in cerebral hemodynamics and oxygenation in the hypercapnia experiment (video played 20x). We performed 100 seconds baseline imaging under normocapnia conditions. Then, we changed the respiratory gas from normal air (with 1.5% isoflurane anesthesia) to a 50%:50% air/CO_2_ mixture to induce hypercapnia for 50 seconds.

**Supplementary Video 2. Cerebrovascular response to hypoxia.**

Hb (Right) and sO_2_ (Left) photoacoustic imaging result of animal under anesthesia, showing changes in cerebral hemodynamics and oxygenation in the hypoxia experiment (video played 20x). We performed 100 seconds baseline imaging under normocapnia conditions. Then, we changed the respiratory gas from normal air (with 1.5% isoflurane anesthesia) to a 50%:50% air/N_2_ mixture to induce hypoxia for 100 seconds.

**Supplementary Video 3. Cerebral imaging in the wakening process.**

Middle: recorded motion of the mouse which wears the headpiece captured by a camera. Hb (Right) and sO_2_ (Left) photoacoustic imaging result captured by the fiberscope, showing changes in cerebral hemodynamics and oxygenation in the awakening process (video played 60x).

**Supplementary Video 4. Hypercapnia in freely behaving healthy mice.**

Middle: recorded motion of the mouse which wears the headpiece captured by a camera. Hb (Right) and sO_2_ (Left) photoacoustic imaging result captured by the fiberscope, showing the hypercapnia-induced cerebral responses in a freely moving healthy mouse (video played 20x). We performed 100 seconds baseline imaging under normocapnia conditions. Then, we changed the respiratory gas from normal air to a 50%:50% air/ CO_2_ mixture to induce hypercapnia for 100 seconds.

**Supplementary Video 5. Hypercapnia in freely behaving obese mice.**

Middle: recorded motion of the mouse which wears the headpiece captured by a camera. Hb (Right) and sO_2_ (Left) photoacoustic imaging result captured by the fiberscope, showing the hypercapnia-induced cerebral responses in a freely moving obese mouse (video played 20x). We performed 100 seconds baseline imaging under normocapnia conditions. Then, we changed the respiratory gas from normal air to a 50%:50% air/ CO_2_ mixture to induce hypercapnia for 100 seconds.

**References**

1. Wang, Q. *et al.* The Allen mouse brain common coordinate framework: a 3D reference atlas. *Cell* **181**, 936-953. e920 (2020).

2. Cao, R. *et al.* Hemodynamic and oxygen-metabolic responses of the awake mouse brain to hypercapnia revealed by multi-parametric photoacoustic microscopy. *Journal of Cerebral Blood Flow & Metabolism* **41**, 2628-2639 (2021).

3. Wenzel, J. *et al.* Impaired endothelium-mediated cerebrovascular reactivity promotes anxiety and respiration disorders in mice. *Proceedings of the National Academy of Sciences* **117**, 1753-1761 (2020).

4. Seker, F. B. *et al.* Neurovascular reactivity in the aging mouse brain assessed by laser speckle contrast imaging and 2-photon microscopy: quantification by an investigator-independent analysis tool. *Frontiers in Neurology* **12**, 745770 (2021).
